# An Anatomically Guided and Optimization-Refined Radiomics Framework for Opportunistic Osteoporosis Assessment from Lumbar Spine MRI

**DOI:** 10.3390/diagnostics16142241

**Published:** 2026-07-17

**Authors:** Akaworn Mahatthanatrakul, Thitiphat Klinsuwan, Rabian Wangkeeree, Artit Laoruengthana

**Affiliations:** 1 Department of Orthopaedics, Faculty of Medicine, Naresuan University, Phitsanulok 65000, Thailand; akawornm@nu.ac.th (A.M.); artitlao@gmail.com (A.L.); 2 Department of Mathematics, Faculty of Science, Naresuan University, Phitsanulok 65000, Thailand; thitiphatarm@gmail.com

**Keywords:** osteoporosis, lumbar spine MRI, radiomics, bone mineral density, texture analysis, optimization, neurodynamic classification

## Abstract

**Background/Objectives:** Osteoporosis is a major contributor to vertebral compression fractures (VCFs) and other skeletal complications, yet quantitative bone mineral density (BMD) assessment using dual-energy X-ray absorptiometry (DEXA) is not routinely available in many spine surgery workflows. This study proposes an anatomically guided and optimization-refined radiomics framework for opportunistic osteoporosis assessment from routine lumbar spine magnetic resonance imaging (MRI). **Methods:** The proposed pipeline employs a hierarchical template-matching strategy to automatically localize the L1–L4 vertebral region, followed by an optimization-based refinement procedure that adapts vertebral regions of interest (ROIs) using intensity, texture, boundary, and geometric constraints. Anatomically consistent ROIs are subsequently used for extraction of handcrafted radiomic descriptors, including statistical, textural, gradient-based, frequency-domain, and shape-related features. The extracted features were evaluated using conventional support vector classification (SVC) and a NeurodynamicSVMRBFTanh classification framework for osteoporosis-related classification. **Results:** Experimental results demonstrated robust and anatomically consistent vertebral localization across heterogeneous lumbar MRI acquisitions. The NeurodynamicSVMRBFTanh framework achieved the best screening-oriented performance, yielding 85.2% classification accuracy and 100.0% sensitivity on an independent test set. In addition, exploratory BMD regression analysis demonstrated the feasibility of estimating DEXA-derived BMD directly from MRI-derived radiomic features, achieving mean absolute percentage errors of approximately 15–20% across lumbar vertebral levels. **Conclusions:** These findings suggest that anatomically guided vertebral radiomics extracted from routine lumbar spine MRI contain clinically meaningful information associated with osteoporosis-related bone quality changes and may provide a practical tool for automated opportunistic osteoporosis assessment in settings where DEXA measurements are unavailable.

## 1. Introduction

Osteoporosis is a major global health disorder characterized by progressive loss of bone mass and deterioration of bone microarchitecture, leading to increased skeletal fragility and fracture risk. More than 37 million fragility fractures occur annually among individuals older than 55 years worldwide [1]. Between 1990 and 2019, mortality attributable to low bone mineral density (BMD) increased by more than 100%, while disability-adjusted life years increased by approximately 94% [2]. Osteoporosis-related fractures, particularly vertebral compression fractures and hip fractures, are associated with substantial morbidity, reduced quality of life, increased healthcare costs, and elevated mortality. In spinal surgery, poor bone quality has also been associated with instrumentation failure, pedicle screw loosening, cage subsidence, pseudoarthrosis, and proximal junctional kyphosis, emphasizing the need for accurate assessment of vertebral bone health.

Dual-Energy X-ray Absorptiometry (DEXA) remains the clinical reference standard for osteoporosis diagnosis and BMD assessment. However, DEXA examinations are not routinely performed for many patients undergoing spinal imaging, resulting in missed opportunities for early osteoporosis detection. In contrast, lumbar magnetic resonance imaging (MRI) is frequently acquired for the evaluation of degenerative spine disease, chronic low back pain, vertebral fractures, and preoperative surgical planning. Consequently, routine lumbar MRI has emerged as a promising source of opportunistic imaging biomarkers that may enable osteoporosis assessment without additional examinations, radiation exposure, or healthcare burden.

The increasing availability of lumbar spine MRI has stimulated growing interest in MRI-based assessment of bone quality and osteoporosis-related changes. Among the most extensively studied MRI-derived biomarkers is the Vertebral Bone Quality (VBQ) score, which is calculated as the ratio between vertebral marrow signal intensity and cerebrospinal fluid (CSF) intensity on T1-weighted MRI [3,4]. Multiple studies have reported significant associations between VBQ and DEXA-derived BMD, fragility fractures, and postoperative spinal complications [3,4,5]. To further enhance predictive performance, Roch et al. [6] demonstrated that combining VBQ measurements derived from T1-, T2-, and STIR-weighted MRI improved osteoporosis assessment compared with single-sequence VBQ measurements, highlighting the complementary information available across different MRI contrasts. In a related effort to simplify clinical implementation, Xue et al. [7] proposed a simplified single-vertebra (T12) VBQ score for opportunistic osteoporosis prediction in patients with degenerative lumbar disease, reporting diagnostic accuracy comparable to multi-level VBQ measurements while requiring only a single manually defined ROI. Beyond VBQ, several additional MRI-derived quantitative biomarkers have been proposed. Saad et al. [8] introduced the M-score, which utilizes vertebral signal intensity characteristics to assess bone quality and demonstrated significant correlations with BMD measurements. More recently, synthetic MRI techniques have enabled quantitative estimation of T1, T2, and proton-density parameters from a single acquisition. Chang et al. [9] reported that synthetic MRI-derived quantitative parameters were significantly associated with lumbar DEXA measurements and could successfully differentiate osteopenic and osteoporotic patients. Collectively, these studies support the feasibility of opportunistic osteoporosis assessment using routinely acquired MRI examinations.

Although intensity-based biomarkers have demonstrated promising diagnostic performance, they primarily rely on manually selected vertebral regions or manually measured signal intensities and may not fully capture the complex structural heterogeneity associated with osteoporosis. Such dependence on manual measurements limits reproducibility, scalability, and integration into routine clinical workflows. To address these limitations, texture analysis and radiomics have increasingly been investigated for quantitative bone quality characterization. Radiomics enables the extraction of high-dimensional image descriptors that characterize intensity distributions, texture patterns, shape properties, structural heterogeneity, gradient information, and frequency-domain characteristics, potentially capturing osteoporosis-related alterations beyond conventional signal-based biomarkers.

Several studies have demonstrated the value of radiomic analysis for bone quality assessment. Maciel et al. [10] reported significant associations between Gray-Level Co-occurrence Matrix (GLCM) texture features and lumbar BMD measurements, suggesting that MRI texture characteristics reflect osteoporosis-related structural alterations. Vara et al. [11] further demonstrated moderate-to-strong correlations between radiomic features extracted from lumbar T1- and T2-weighted MRI and DEXA-derived BMD values. More recently, Liu et al. [12] showed that radiomic features combined with machine learning achieved strong performance for opportunistic osteoporosis screening using vertebral CT images. In parallel, a small number of studies have begun to automate the radiomics pipeline itself. Zhao et al. [13] combined deep-learning-based vertebral segmentation with radiomic feature extraction from a modified Dixon MRI sequence to classify abnormal bone density and osteoporosis against a quantitative CT reference. Similarly, Galbusera et al. [14] integrated MRI-derived radiomics and deep learning with radiographic measurements and clinical metadata to both estimate BMD and classify osteopenia/osteoporosis. These studies demonstrate that automated vertebral segmentation can be successfully coupled with radiomic or deep-learning-based analysis; however, neither reports feature-level interpretability, so the relative contribution of different radiomic feature categories to the underlying predictions remains unexplained.

Despite these encouraging findings, a common limitation across most VBQ-, M-score-, and radiomics-based approaches is their reliance on manually selected vertebral regions of interest (ROIs). This limitation is particularly important for radiomics because extracted features are highly sensitive to ROI placement and boundary definition. Small variations in vertebral localization may substantially alter feature values and consequently affect downstream predictive performance. Manual ROI selection also introduces observer variability, limits scalability, and presents a substantial obstacle to large-scale opportunistic screening using routine clinical imaging examinations. Even the few recent pipelines that eliminate manual ROI selection through deep-learning-based segmentation [13,14] operate as opaque predictors, offering no insight into which image characteristics drive their classification or regression outputs. Therefore, anatomically consistent and reproducible vertebral localization, combined with interpretable feature analysis, represents a critical and still largely unaddressed prerequisite for reliable MRI-based osteoporosis assessment.

Automated vertebral localization remains a challenging medical image analysis problem because of substantial variability in spinal curvature, vertebral morphology, image contrast, field-of-view coverage, and acquisition protocols. Although manual ROI selection can achieve acceptable performance in controlled research settings, it constrains the deployment of fully automated opportunistic screening systems in routine clinical practice. Consequently, there remains a need for automated frameworks capable of robustly identifying anatomically consistent vertebral regions while maintaining reproducible feature extraction across heterogeneous MRI examinations, and while providing transparency into the imaging characteristics underlying model predictions.

To address these challenges, this study proposes a fully automated and anatomically guided radiomics framework for opportunistic osteoporosis assessment from routine lumbar spine MRI. Unlike conventional MRI-derived biomarkers such as VBQ and M-score, which rely on manual vertebral localization and signal intensity measurements, the proposed framework performs automated vertebral localization, optimization-based ROI refinement, anatomically consistent radiomic feature extraction, and machine learning-based osteoporosis assessment within a unified pipeline. Vertebral regions are first identified using a hierarchical localization strategy and subsequently refined through an optimization procedure incorporating image intensity, anatomical boundary, and geometric constraints. Radiomic descriptors encompassing statistical, textural, structural, shape-related, and frequency-domain characteristics are then extracted and evaluated using both conventional Support Vector Classification (SVC) and a neurodynamic kernel-based classification framework. In addition, exploratory BMD regression analysis is performed to investigate the feasibility of estimating DEXA-derived BMD directly from MRI-derived radiomic features.

The main contributions of this work are fourfold:

1. The introduction of a fully automated anatomically guided vertebral localization and optimization-based ROI refinement framework for lumbar MRI.
2. The development of an anatomically consistent multi-domain radiomics pipeline for opportunistic osteoporosis assessment.
3. The evaluation of the proposed framework for both osteoporosis classification and exploratory BMD estimation using routine clinical MRI examinations.
4. The incorporation of feature-group importance analysis using permutation importance and Shapley values to identify the radiomic feature categories that drive osteoporosis classification, thereby improving the interpretability of the proposed framework.

By eliminating manual vertebral ROI selection while providing anatomically consistent feature extraction and model interpretability, the proposed framework extends existing MRI-based osteoporosis assessment from manually derived intensity biomarkers toward a fully automated, explainable radiomics pipeline. This design has the potential to improve reproducibility, reduce observer variability, and facilitate opportunistic osteoporosis screening within routine clinical radiology workflows.

## 2. Materials and Methods

This study developed a fully automated framework for opportunistic osteoporosis assessment from routine lumbar spine MRI. The proposed methodology consists of four main stages: (1) hierarchical localization of the lumbar spine and vertebral bodies, (2) optimization-based vertebral ROI refinement, (3) radiomic feature extraction and feature preprocessing, and (4) machine learning-based osteoporosis classification and exploratory bone mineral density (BMD) prediction.

### 2.1. Dataset

This retrospective study used lumbar spine magnetic resonance imaging (MRI) examinations and corresponding dual-energy X-ray absorptiometry (DEXA) measurements collected from the Department of Orthopedics, Naresuan University Hospital, Thailand, under institutional ethical approval. All patient identifiers were removed before analysis in accordance with patient privacy and data protection requirements. Bone mineral density measurements were acquired using a Hologic DEXA scanner (Discovery W model), which served as the reference standard for osteoporosis assessment.

The dataset comprised 167 subjects with lumbar spine MRI examinations acquired using a Philips Ingenia 1.5T MRI system (Philips Medical Systems Nederland B.V., Best, The Netherlands) system and clinically acquired DEXA measurements. DEXA-derived measurements were used as reference labels for osteoporosis classification and exploratory bone mineral density (BMD) prediction, as DEXA remains the clinical reference standard for osteoporosis diagnosis and quantitative BMD assessment.

Three routinely acquired sagittal MRI sequences were considered in this study: T1-weighted (T1W), T2-weighted (T2W), and Short Tau Inversion Recovery (STIR). These sequences provide complementary information regarding vertebral marrow composition, anatomical structure, tissue heterogeneity, and fluid-sensitive contrast. T1-weighted imaging is sensitive to marrow fat content, T2-weighted imaging provides anatomical visualization, and STIR imaging suppresses fat signal while emphasizing fluid-sensitive abnormalities. Each available sequence was processed independently using the same automated localization, ROI refinement, and radiomic feature extraction pipeline.

MRI acquisition characteristics are summarized in Table 1. Images were acquired with a slice thickness of 4.0 mm, spacing between slices of 4.4 mm, and in-plane pixel spacing ranging from 0.3472×0.3472 to 0.5294×0.5294 mm. Image dimensions ranged from 400×400 to 1024×1024 pixels depending on acquisition and reconstruction settings. The target anatomical region comprised lumbar vertebral levels L1–L4, which were used for vertebral localization, ROI refinement, and radiomic feature extraction.

Cases were included if they met the following criteria: (1) availability of sagittal lumbar spine MRI containing the target lumbar vertebral region; (2) availability of corresponding DEXA measurements; (3) MRI and DEXA examinations acquired within a 12-month interval; and (4) sufficient image quality for anatomical localization and radiomic analysis. After applying these criteria, 167 subjects were retained for analysis.

Reference labels were derived from DEXA T-scores according to the World Health Organization (WHO) diagnostic criteria. Subjects were categorized into three diagnostic groups: Normal, Osteopenia, and Osteoporosis. The class distribution is summarized in Table 2.

Although the original dataset was organized according to the three WHO-defined diagnostic categories, the primary objective of this study was automated identification of osteoporosis from routine lumbar MRI. Therefore, the main classification task was formulated as a binary screening problem by combining the Normal and Osteopenia groups into a non-osteoporotic category and treating Osteoporosis as the positive class. This formulation reflects a clinically relevant opportunistic screening scenario in which subjects with osteoporosis can be automatically identified for further clinical evaluation. The resulting binary class distribution is summarized in Table 3.

In addition to diagnostic class labels, the corresponding DEXA examinations provided quantitative lumbar spine BMD measurements, including vertebral-level measurements from L1 to L4. These measurements were used as reference values in exploratory regression experiments to investigate the feasibility of estimating DEXA-derived BMD directly from MRI-derived radiomic features.

### 2.2. Overview of the Proposed Framework

The proposed framework aims to perform opportunistic osteoporosis assessment from routine lumbar spine magnetic resonance imaging (MRI) through anatomically guided vertebral radiomic analysis. The overall pipeline consists of four major stages: (1) image preprocessing and standardization, (2) anatomically guided vertebral localization and optimization-based ROI refinement, (3) multi-scale radiomic feature extraction, and (4) osteoporosis-related assessment using machine learning models.

First, lumbar spine MRI scans are preprocessed to reduce inter-subject variability and improve anatomical consistency across the dataset. The preprocessing stage includes image resizing, intensity normalization, and automatic background cropping to standardize the field of view while preserving clinically relevant vertebral structures.

Next, a hierarchical localization framework is employed to automatically identify the lumbar vertebral region containing the L1–L4 vertebrae. The localization process consists of two stages: coarse localization of the lumbar spine using a context-aware anatomical template, followed by fine detection of individual vertebral levels. To improve anatomical consistency and reduce boundary variability, each detected vertebral region is subsequently refined using an optimization-based ROI extraction framework that integrates photometric, structural, and geometric constraints.

Following ROI refinement, a comprehensive set of handcrafted radiomic features is extracted from each vertebral body. The extracted features encompass complementary image characteristics, including first-order intensity statistics, texture descriptors, structural and gradient-based features, frequency-domain representations, and shape-related features. Feature vectors from the individual vertebral levels are subsequently aggregated to form a patient-level radiomic representation.

To reduce feature redundancy and mitigate overfitting associated with high-dimensional radiomic data, all extracted features are standardized and transformed using principal component analysis (PCA). The resulting low-dimensional feature representation is then used for osteoporosis-related assessment.

Finally, the discriminative capability of the proposed vertebral radiomic representation is evaluated using both conventional Support Vector Classification (SVC) and the NeurodynamicSVMRBFTanh framework. In addition, exploratory regression experiments are performed to investigate the feasibility of estimating DEXA-derived bone mineral density (BMD) directly from MRI-derived vertebral radiomic features. The overall objective is to determine whether anatomically consistent vertebral radiomics extracted from routine lumbar spine MRI contain clinically meaningful information associated with osteoporosis-related bone quality changes.

### 2.3. Image Preprocessing

Lumbar spine MRI examinations were acquired using different imaging protocols and reconstruction settings, resulting in variations in image dimensions, spatial resolution, and intensity distributions across subjects. To reduce inter-subject variability and improve the robustness of subsequent anatomical localization and radiomic analysis, a standardized preprocessing pipeline was applied to all images. The preprocessing stage consisted of image resizing, intensity normalization, and automatic background cropping.

#### 2.3.1. Resizing to Uniform Dimensions

Because the original MRI scans exhibited varying spatial dimensions, all images were resampled to a common spatial resolution prior to analysis. Let the original image be denoted as I(x,y) with dimensions (H,W). Each image was resized to a standardized resolution of 1024×1024 pixels using bilinear interpolation:

(1) $$I^{\prime }\left(x,y\right)=R\left(I\left(x,y\right);1024,1024\right)$$

where R(·) denotes the resampling operator. Standardizing image dimensions facilitates template-based localization and ensures that anatomical structures occupy comparable spatial scales across the dataset.

#### 2.3.2. Intensity Normalization

MRI signal intensity lacks a standardized physical scale and may vary substantially across acquisitions due to differences in scanner settings, patient characteristics, and reconstruction procedures. To improve intensity consistency while preserving relative tissue contrast, min–max normalization was applied:

(2) $$I^{\prime }\left(x,y\right)=\frac{I\left(x,y\right)-I_{\min}}{I_{\max}-I_{\min}}$$

where $I_{\min}$ and $I_{\max}$ denote the minimum and maximum pixel intensities within the image. This transformation maps all pixel values into the range [0, 1] while maintaining the relative intensity relationships between anatomical structures.

#### 2.3.3. Background Cropping and Anatomical ROI Preservation

Although the lumbar spine occupies only a portion of the acquired field of view, the original MRI scans often contain large background regions that do not contribute meaningful anatomical information. To reduce irrelevant image content and improve computational efficiency, an automatic cropping procedure was performed.

A binary foreground mask was first generated using intensity thresholding:

(3) $$B\left(x,y\right)=\left\{\begin{array}{cc} 1, & \mathrm{if}\;I(x,y)>\tau \\ 0 & \mathrm{otherwise} \end{array}\right.$$

where τ denotes an empirically determined intensity threshold. The largest connected foreground component was subsequently identified, and the smallest bounding box enclosing this region was used to crop the image.

**Figure 1:**
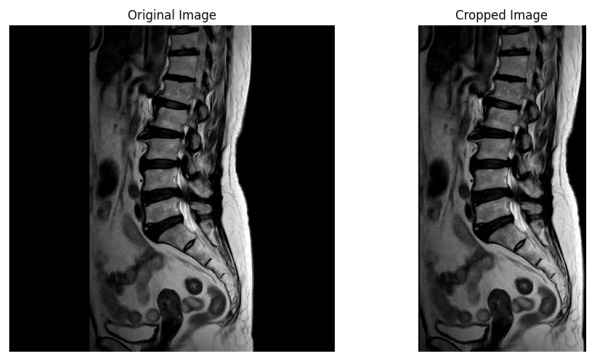
Preprocessing step showing the original MRI image and the automatically cropped image used for subsequent analysis.

This procedure preserves the vertebral column and surrounding anatomical structures while removing large background areas that may negatively influence subsequent template matching and feature extraction stages. An example of the cropping process is illustrated in Figure 1.

Following preprocessing, all MRI scans were standardized with respect to image dimensions, intensity range, and anatomical field of view. These standardized images served as the input for the anatomically guided vertebral localization and optimization-based ROI refinement stages of the proposed framework.

### 2.4. Anatomically Guided Vertebral Localization

Accurate identification of vertebral anatomy is a fundamental prerequisite for reliable radiomic analysis. Because radiomic features are highly sensitive to ROI placement and anatomical consistency, inaccurate localization may introduce substantial variability in feature extraction and subsequently affect osteoporosis assessment performance. In lumbar spine MRI, vertebral localization is particularly challenging due to inter-subject anatomical variation, spinal curvature, degenerative changes, heterogeneous image contrast, and variations in image acquisition protocols.

**Figure 2:**
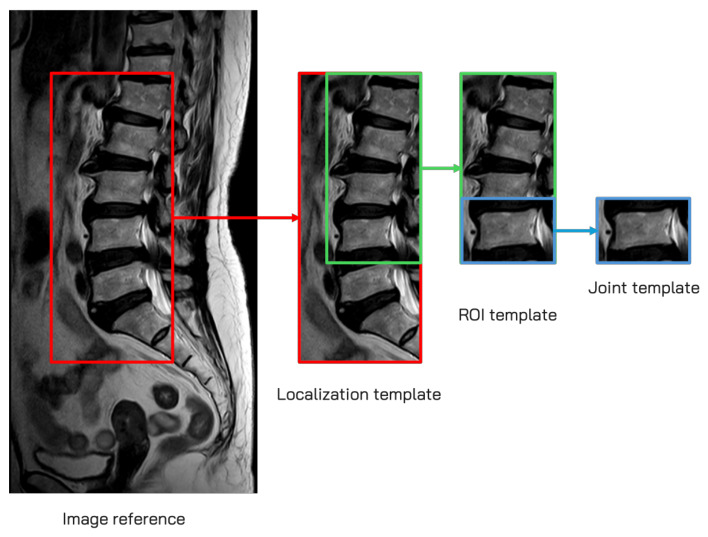
Illustration of the hierarchical template creation pipeline. A block template covering T12–L5 (red) was first used for localization, followed by extraction of the L1–L4 ROI (green) and finally smaller joint templates (blue).

To address these challenges, we developed an anatomically guided vertebral localization framework that combines hierarchical template matching with anatomical constraints. Rather than directly detecting individual vertebral levels across the entire image, the proposed approach first localizes the lumbar spinal region containing the target vertebrae and subsequently performs fine-grained detection of individual vertebral levels. This hierarchical strategy reduces the search space, improves robustness to anatomical variability, and provides anatomically plausible initialization for the subsequent ROI refinement stage. The overall concept of template construction and hierarchical matching is illustrated in Figure 2.

#### 2.4.1. Lumbar Region Localization

The objective of the first localization stage is to identify the lumbar spinal region containing the target vertebral levels (L1–L4). Direct localization of individual vertebrae can be challenging because local vertebral appearance may vary considerably between patients. Therefore, instead of directly targeting L1–L4, a larger anatomical template spanning the T12–L5 region was employed.

This extended anatomical context incorporates multiple vertebral bodies and intervertebral structures, allowing the localization process to exploit global spinal organization rather than relying solely on local image appearance. As a result, the method is more robust to spinal curvature, anatomical variability, and intensity heterogeneity commonly observed in lumbar MRI.

Let *I* denote the input image and $T_{\mathrm{block}}$ the anatomical block template. A similarity score map S(x,y) is computed by sliding the template across the image. Multiple similarity measures were evaluated to characterize different matching behaviors.

Sum of Squared Differences (SSD)

(4) $$S_{\mathrm{SSD}}\left(x,y\right)=-\sum_{i,j}{\left(I\left(x+i,y+j\right)-T_{\mathrm{block}}\left(i,j\right)\right)}^{2},$$

where the negative sign is applied so that higher values correspond to better matches.

Normalized Cross-Correlation (NCC)

(5) $$S_{\mathrm{NCC}}\left(x,y\right)=\frac{\sum_{i,j}\left(I\left(x+i,y+j\right)-\mu_{I}\right)\left(T_{\mathrm{block}}\left(i,j\right)-\mu_{T}\right)}{\sqrt{\sum_{i,j}{\left(I\left(x+i,y+j\right)-\mu_{I}\right)}^{2}};\sqrt{\sum_{i,j}{\left(T_{\mathrm{block}}\left(i,j\right)-\mu_{T}\right)}^{2}}},$$

where $\mu_{I}$ and $\mu_{T}$ denote the mean intensities of the image patch and template, respectively.

Structural Similarity Index (SSIM)

(6) $$S_{\mathrm{SSIM}}\left(x,y\right)=\frac{\left(2\mu_{I}\mu_{T}+C_{1}\right)\left(2\sigma_{IT}+C_{2}\right)}{\left(\mu_{I}^{2}+\mu_{T}^{2}+C_{1}\right)\left(\sigma_{I}^{2}+\sigma_{T}^{2}+C_{2}\right)},$$

where $\sigma_{I}^{2}$ and $\sigma_{T}^{2}$ are variances, $\sigma_{IT}$ is the covariance between the image patch and template, and $C_{1}$ and $C_{2}$ are stabilization constants.

Mutual Information (MI)

(7) $$S_{\mathrm{MI}}\left(x,y\right)=\sum_{a,b}p_{IT}\left(a,b\right)\log\frac{p_{IT}\left(a,b\right)}{p_{I}\left(a\right),p_{T}\left(b\right)},$$

where $p_{IT}\left(a,b\right)$ is the joint probability distribution of intensities in the image patch and template, and $p_{I}$ and $p_{T}$ are their marginal distributions.

The optimal location is determined as

(8) $$\left(x^{*},y^{*}\right)=\arg\max_{x,y}S\left(x,y\right),$$

where S(x,y) corresponds to the selected similarity metric.

**Figure 3:**
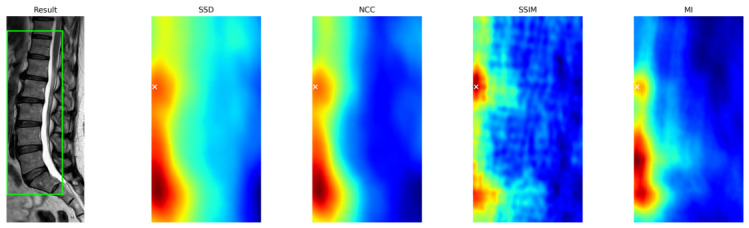
Coarse localization of the lumbar spine region using a context-aware block template (T12–L5). Score maps from four similarity metrics (SSD, NCC, SSIM, and MI) are shown. Warmer colors indicate higher similarity scores, and the white cross marks the selected optimal location. The green bounding box in the Result panel represents the extracted candidate region containing L1–L4.

**Figure 4:**
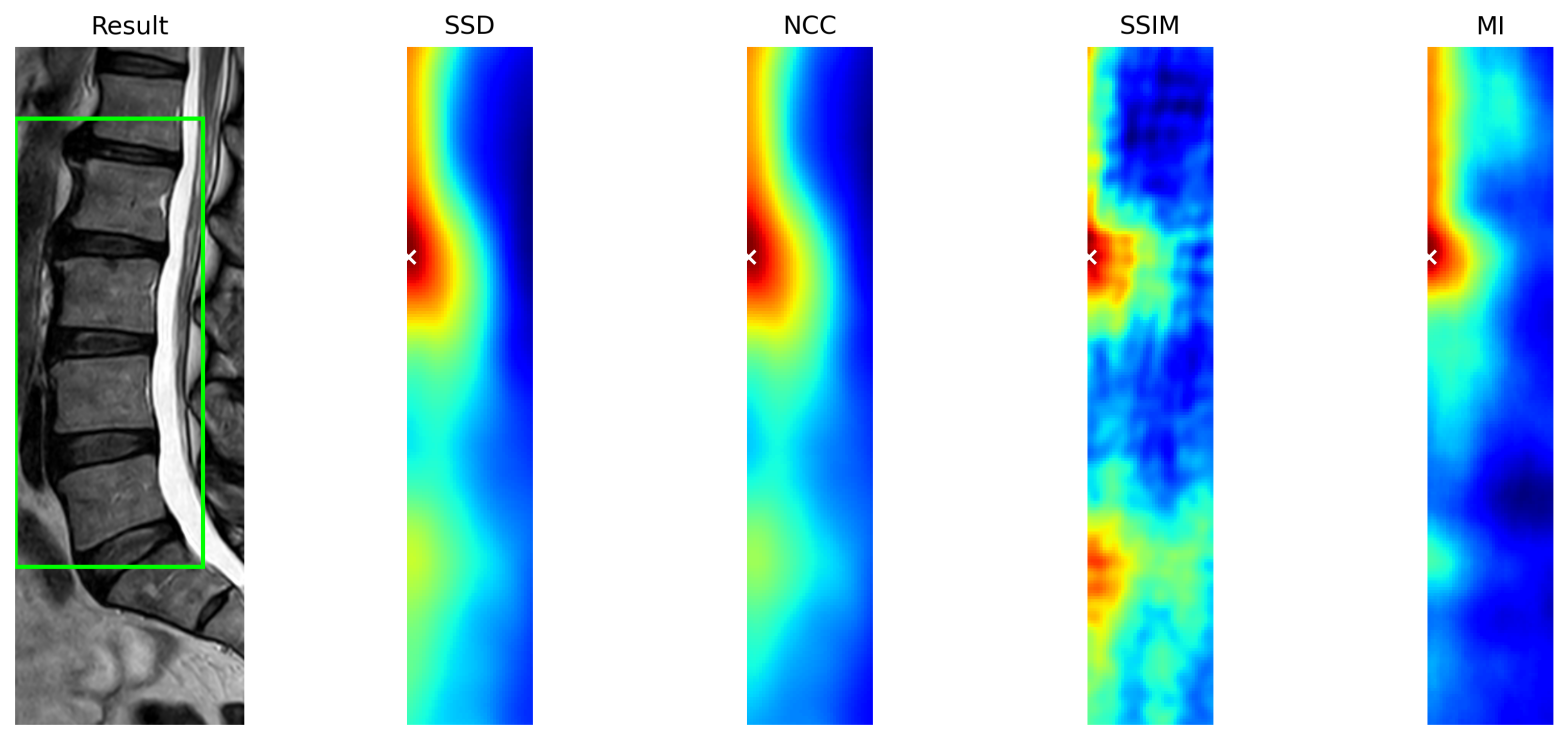
Refinement of the localized lumbar region using a region-of-interest (ROI) template. The input to this stage is the cropped region obtained from Figure 3. Score maps from SSD, NCC, SSIM, and MI are shown for comparison. Warmer colors indicate higher similarity scores, and the white cross denotes the selected optimal location. The green bounding box in the Result panel indicates the refined region for subsequent analysis.

Figure 3 and Figure 4 illustrate representative examples of the localization process. The first stage identifies the lumbar spinal block using an extended anatomical template, while the second stage refines localization within the extracted candidate region. Although different similarity measures exhibit distinct response characteristics, all methods consistently emphasize the lumbar spinal column as the dominant anatomical structure. These results demonstrate that incorporating extended anatomical context provides a reliable initialization for subsequent vertebral-level detection.

The output of this stage is a localized lumbar region containing the target vertebral levels, which serves as the search space for individual vertebral detection.

#### 2.4.2. Individual Vertebral Detection

Following lumbar region localization, the second stage aims to identify the individual vertebral levels within the extracted lumbar region. Because vertebral bodies are organized along a continuous anatomical trajectory, vertebral detection should not be treated as an independent local matching problem. Instead, anatomical relationships between neighboring vertebrae can be exploited to improve detection robustness and suppress anatomically implausible candidates.

To achieve this, the proposed framework combines local template responses with global spinal alignment estimation. For each vertebral template $T_{Lk}$, a similarity map $S_{k}\left(x,y\right)$ is first computed within the localized lumbar region. Rather than selecting vertebral candidates solely based on local maxima, a global spinal trajectory is estimated from the complete similarity distribution and subsequently used as an anatomical prior during candidate selection.

For each image row *y*, a robust horizontal center c(y) is computed as a weighted average of high-confidence responses:

(9) $$c\left(y\right)=\frac{\sum_{x}x\cdot \phi !\left(S_{k}\left(x,y\right)\right)}{\sum_{x}\phi !\left(S_{k}\left(x,y\right)\right)},$$

where ϕ(·) suppresses weak responses while emphasizing high-confidence regions. Specifically,

(10) $$\phi \left(s\right)=\left\{\begin{array}{cc} s^{p_{\mathrm{row}}}, & s\geq q_{\mathrm{row}},\\ 0, & \mathrm{otherwise}, \end{array}\right.$$

where $q_{\mathrm{row}}$ denotes the row-wise response quantile threshold and $p_{\mathrm{row}}$ controls response amplification.

The resulting row-wise centers are subsequently smoothed using moving-average regularization and Gaussian filtering along the vertical direction before fitting a weighted polynomial curve

(11) x=f(y),

which models the global spinal alignment.

Based on the fitted curve, a spatial prior map is constructed as

(12) $$W\left(x,y\right)=\exp\left(-\frac{{\left(x-f\left(y\right)\right)}^{2}}{2\sigma_{x}^{2}}\right),$$

where $\sigma_{x}$ controls the width of the anatomical prior around the estimated spinal trajectory.

The similarity map is then refined according to

(13) $$S_{k}^{\prime }\left(x,y\right)=S_{k}\left(x,y\right)\left[(1-\alpha )+\alpha W(x,y)\right],$$

where α controls the strength of anatomical guidance.

Candidate vertebral locations are extracted from the refined similarity map using a statistical threshold

(14) $$\tau =\mu_{S^{\prime }}+k\sigma_{S^{\prime }},$$

where $\mu_{S^{\prime }}$ and $\sigma_{S^{\prime }}$ denote the mean and standard deviation of the refined similarity scores, respectively. To avoid multiple detections of the same vertebral body, non-maximum suppression is applied using a minimum peak separation distance proportional to the vertebral template height.

Rather than selecting vertebral candidates independently, a combinatorial optimization strategy is subsequently employed. Let

(15) $$P={\left(x_{i},y_{i}\right)}_{i=1}^{N}$$

denote the candidate peak locations with normalized similarity scores ${\tilde{S}}_{i}$. The optimal vertebral configuration is determined by minimizing

(16) $$L=\lambda_{\mathrm{line}}E_{\mathrm{line}}+\lambda_{\mathrm{spacing}}E_{\mathrm{spacing}}+\lambda_{\mathrm{spread}}E_{\mathrm{spread}}+\lambda_{\mathrm{score}}E_{\mathrm{score}},$$

where

(17) $$E_{\mathrm{line}}=\sqrt{\frac{1}{K}\sum_{i=1}^{K}{\left(x_{i}-f\left(y_{i}\right)\right)}^{2}},$$

measures the deviation of candidate vertebral locations from the estimated spinal trajectory,

(18) $$E_{\mathrm{spacing}}=\mathrm{std}\left(\Delta y\right),$$

penalizes inconsistent inter-vertebral spacing,

(19) $$E_{\mathrm{spread}}=-\mathrm{mean}\left(\Delta y\right),$$

encourages sufficient separation between consecutive vertebral levels, and

(20) $$E_{\mathrm{score}}=-\frac{1}{K}\sum_{i=1}^{K}{\tilde{S}}_{i},$$

encourages candidate configurations supported by strong template-matching responses. Here, Δy denotes the vertical distances between consecutive vertebral levels.

The parameter values reported in Table 4 were determined empirically using the training cohort and subsequently fixed for all experiments. In particular, α=1.0 fully incorporates the anatomical prior during candidate refinement, while the optimization weights balance spinal alignment consistency, inter-vertebral spacing regularity, vertebral coverage, and template-matching confidence.

The optimal vertebral candidates are subsequently converted into bounding regions using the corresponding template dimensions. By jointly integrating local image evidence and global anatomical structure, the proposed framework produces anatomically coherent vertebral localization even in the presence of spinal curvature, image noise, and intensity variability. These localized vertebral regions provide the initialization for the optimization-based ROI refinement stage described in the following section.

### 2.5. Optimization-Based Vertebral ROI Refinement

Although the hierarchical localization framework provides reliable identification of vertebral levels, the resulting bounding regions remain approximate and do not necessarily correspond to anatomically consistent vertebral boundaries. Such variability can substantially influence radiomic feature extraction because many texture, structural, and frequency-based descriptors are highly sensitive to ROI definition. Consequently, achieving anatomically consistent vertebral ROIs is critical for obtaining reproducible and clinically meaningful radiomic representations.

**Figure 5:**
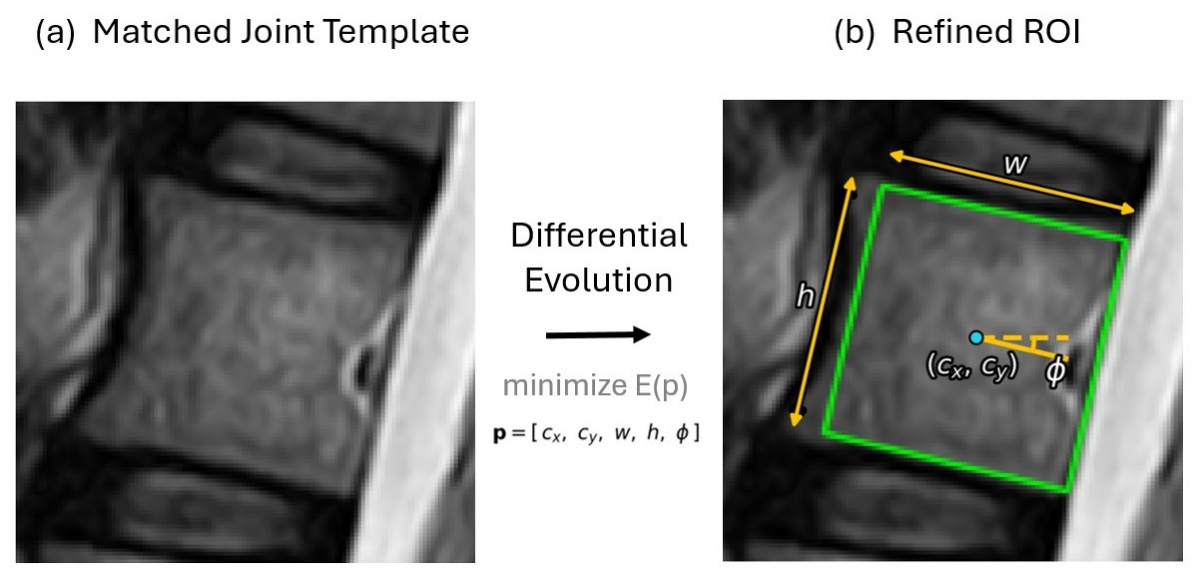
Optimization-based vertebral ROI refinement. (**a**) Approximate vertebral region obtained from the hierarchical matched-template localization, which may be misaligned or include surrounding structures. (**b**) Refined ROI after optimization: Differential Evolution adjusts the rotated-rectangle parameters $p=[c_{x},c_{y},w,h,\phi ]$—center $\left(c_{x},c_{y}\right)$, width *w*, height *h*, and rotation angle ϕ—to minimize the composite energy E(p), yielding a boundary that is closely aligned with the vertebral body.

To address this challenge, we introduce an optimization-based vertebral ROI refinement framework that adaptively adjusts the position, orientation, and dimensions of each vertebral region according to image appearance, structural boundaries, and anatomical priors. Unlike conventional fixed-window approaches, the proposed method explicitly seeks an ROI configuration that maximizes anatomical consistency while minimizing the inclusion of irrelevant surrounding structures. As this refinement constitutes the core methodological contribution of the proposed pipeline, its overall flow is summarized in Figure 5: an approximate region obtained from the matched joint template (a) is progressively adjusted through Differential Evolution into an anatomically aligned ROI (b), parameterized as a rotated rectangle.

By integrating photometric consistency, structural edge information, geometric regularization, and anatomical constraints within a unified energy formulation, the method generates robust vertebral representations suitable for reproducible radiomic analysis across heterogeneous MRI acquisitions. Extracting radiomic features directly from the unrefined matched-template region would inevitably incorporate signal from adjacent non-vertebral structures; the proposed refinement instead guarantees that the analyzed region reflects only vertebral attributes. The qualitative refinement steps and the corresponding convergence analysis are presented in Section 3.

As illustrated in Figure 5b, each vertebral ROI is represented as a rotated rectangle parameterized by

$$p=[c_{x},c_{y},w,h,\phi ],$$

where $c_{x}$ and $c_{y}$ denote the rectangle center coordinates, *w* and *h* represent the rectangle width and height, and ϕ denotes the rotation angle. The optimal ROI configuration is obtained by minimizing a composite energy function that balances appearance fidelity, structural alignment, and anatomical plausibility:

(21) $$E\left(p\right)=\alpha E_{\mathrm{data}}+\beta E_{\mathrm{spread}}-\gamma E_{\mathrm{border}}+\delta E_{\mathrm{aspect}}+\eta E_{\mathrm{pos}}+\epsilon E_{\mathrm{leak}}+\zeta_{d}E_{\mathrm{dark}}+\zeta_{b}E_{\mathrm{bright}}.$$

The selected energy terms were designed to balance competing objectives during vertebral localization. While the homogeneity term promotes internally consistent marrow appearance, the boundary term encourages alignment with vertebral edges. Geometric and positional regularization terms prevent anatomically implausible solutions, whereas occupancy penalties discourage inclusion of background regions and intensity outliers frequently encountered in lumbar MRI. Each energy term captures a complementary aspect of vertebral anatomy and image appearance, allowing the optimization process to balance local image evidence against global anatomical constraints.

(1) Data consistency term $E_{\mathrm{data}}$

This term encourages the average signal intensity within the ROI to remain consistent with the expected vertebral marrow appearance. By promoting agreement with characteristic vertebral tissue intensity, the optimization process favors regions likely to correspond to vertebral anatomy rather than surrounding soft tissues.

(22) $$E_{\mathrm{data}}=\left|\frac{1}{\left|\Omega \right|}\sum_{(x,y)\in \Omega }I\left(x,y\right)-I_{t}\right|.$$

(2) Homogeneity term $E_{\mathrm{spread}}$

Vertebral marrow generally exhibits relatively homogeneous signal characteristics compared with surrounding anatomical structures. To promote internal consistency within the ROI, a robust intensity dispersion measure based on the median absolute deviation (MAD) is employed:

(23) $$E_{\mathrm{spread}}=\mathrm{median}{\left(|I(x,y)-\mathrm{median}(I)|\right)}_{(x,y)\in \Omega }.$$

(3) Boundary strength term $E_{\mathrm{border}}$

Anatomically meaningful vertebral boundaries are typically associated with elevated image gradients. This term encourages ROI alignment with visible vertebral margins by maximizing the average gradient magnitude along the ROI boundary region:

(24) $$E_{\mathrm{border}}=\frac{1}{\left|R\right|}\sum_{(x,y)\in R}G\left(x,y\right).$$

Because stronger gradients indicate better boundary alignment, this term is subtracted in the overall energy formulation.

(4) Aspect ratio regularization $E_{\mathrm{aspect}}$

To preserve anatomically plausible vertebral geometry, the ROI aspect ratio is constrained to remain close to an expected vertebral shape:

(25) $$E_{\mathrm{aspect}}={\left(\frac{a}{a_{0}}-1\right)}^{2}.$$

This regularization prevents unrealistic ROI elongation or compression during optimization.

(5) Positional prior $E_{\mathrm{pos}}$

Because vertebral bodies are arranged along a continuous spinal trajectory, large deviations from the estimated spinal midline are anatomically unlikely. This term softly constrains the ROI center to remain near the expected vertebral alignment:

(26) $$E_{\mathrm{pos}}={\left(c_{x}-x_{m}\right)}^{2}.$$

(6) Leakage penalty $E_{\mathrm{leak}}$

To prevent anatomically invalid solutions, a leakage penalty is introduced whenever portions of the ROI extend beyond the image boundaries. This term encourages all candidate ROIs to remain fully contained within the valid image domain.

(7) Occupancy penalties $E_{\mathrm{dark}}$ and $E_{\mathrm{bright}}$

Lumbar MRI frequently contains background regions, imaging artifacts, and highly heterogeneous intensity structures that are not representative of vertebral anatomy. To reduce contamination from such regions, occupancy penalties are applied based on the proportion of extremely dark or extremely bright pixels within the ROI:

(27) $$E_{\mathrm{dark}}={\left(\frac{|I\left(x,y\right)<T_{d}|}{\left|\Omega \right|}\right)}^{2},\;E_{\mathrm{bright}}={\left(\frac{|I\left(x,y\right)>T_{b}|}{\left|\Omega \right|}\right)}^{2}.$$

These penalties discourage inclusion of background regions, imaging artifacts, and overexposed structures.

The optimization of Equation (21) was performed using Differential Evolution (DE) Storn and Price [15], a population-based global optimization algorithm particularly suitable for non-convex and non-differentiable objective functions. The optimization hyperparameters and refinement settings adopted throughout this study are listed in Table 5. At each generation, candidate ROI configurations evolve through stochastic mutation and crossover operations, with selection driven by the composite energy value. In practice, Differential Evolution consistently converged to stable ROI configurations within the predefined optimization budget.

The resulting refined ROIs exhibit improved anatomical consistency compared with the initial template-based detections. By jointly integrating photometric characteristics, structural edge information, geometric regularization, and anatomical priors, the proposed refinement framework produces vertebral representations that are well suited for subsequent radiomic feature extraction. This refinement process is particularly important because radiomic reproducibility depends strongly on ROI consistency, and even small boundary variations may substantially alter extracted feature values.

### 2.6. Multi-Scale Radiomic Feature Extraction

Following optimization-based vertebral ROI refinement, a comprehensive radiomic feature extraction framework was applied to characterize vertebral image properties associated with osteoporosis-related bone quality changes. The objective of this stage was to transform each anatomically refined vertebral ROI into a quantitative representation capable of capturing complementary aspects of marrow composition, trabecular organization, structural heterogeneity, and vertebral morphology.

Osteoporosis affects bone tissue across multiple spatial scales, ranging from diffuse changes in bone marrow composition to localized deterioration of the trabecular network. These pathological alterations manifest through changes in signal intensity, spatial heterogeneity, local structural organization, frequency characteristics, and, in more advanced cases, vertebral morphology. Consequently, no single category of radiomic descriptors is expected to comprehensively characterize osteoporosis-related imaging changes. To capture these complementary properties, the proposed framework extracts five groups of handcrafted radiomic features: (1) statistical features, which summarize the distribution of pixel intensities and reflect global marrow signal characteristics; (2) texture features, which quantify local spatial heterogeneity and trabecular organization through gray-level relationships and local intensity patterns; (3) structural features, which characterize edge information and local anatomical organization using gradient-based descriptors; (4) frequency-domain features, which capture multi-scale image patterns and spatial frequency content associated with trabecular architecture; and (5) shape features, which describe the geometric characteristics of the segmented vertebral body. Collectively, these feature groups provide complementary information spanning global intensity distributions, local texture, structural organization, multi-scale frequency content, and vertebral morphology.

For each vertebral level (L1–L4), radiomic features were extracted independently from the corresponding optimization-refined ROI. The resulting vertebral feature vectors were subsequently concatenated to form a patient-level radiomic representation for downstream osteoporosis classification. The relative contribution of each radiomic feature group was further investigated through feature-group importance analysis, as presented in Section 3.3.3, to identify which image characteristics contribute most significantly to osteoporosis discrimination.

#### 2.6.1. Region of Interest (ROI) Definition

For each vertebra, feature extraction was restricted to the optimized region defined by its binary mask M(x,y). Let I(x,y) denote the corresponding grayscale MRI image. The region of interest (ROI) is defined as:

(28) $$R=\left\{I(x,y)|M(x,y)=1\right\}$$

All feature computations were constrained to this anatomically refined ROI to ensure vertebral specificity and minimize contamination from surrounding anatomical structures.

#### 2.6.2. Intensity Features

First-order intensity features describe the statistical distribution of pixel intensities within the vertebral ROI without considering spatial relationships between neighboring pixels. These descriptors provide information regarding vertebral marrow composition and global signal characteristics, both of which may be influenced by osteoporosis-related marrow fat infiltration and bone demineralization.

To characterize the intensity distribution, measures of central tendency, dispersion, distribution shape, and information content were extracted, including mean intensity, standard deviation, minimum intensity, maximum intensity, entropy, skewness, and kurtosis:

(29) $$\begin{array}{ccc} \mu & =\frac{1}{\left|R\right|}\sum_{i=1}^{\left|R\right|}R_{i} & \left(\mathrm{Mean}\right) \end{array}$$

(30) $$\begin{array}{ccc} \sigma & =\sqrt{\frac{1}{\left|R\right|}\sum_{i=1}^{\left|R\right|}{\left(R_{i}-\mu \right)}^{2}} & (\mathrm{Standard}\;\mathrm{Deviation}) \end{array}$$

(31) $$\begin{array}{ccc} \min(R),\max(R) & \; & (\mathrm{Min},\;\mathrm{Max}\;\mathrm{Intensity}) \end{array}$$

(32) $$\begin{array}{ccc} H & =-\sum_{k=1}^{K}p_{k}\log\left(p_{k}+\epsilon \right) & (\mathrm{Shannon}\;\mathrm{Entropy}) \end{array}$$

(33) $$\begin{array}{cc} \mathrm{Skewness} & =\frac{1}{\left|R\right|}\sum_{i=1}^{\left|R\right|}{\left(\frac{R_{i}-\mu }{\sigma }\right)}^{3}\;\;\;\;\;\;\;\;\;\;\;\;\;\;\;\;\;\;\;\;\;\;\;\;\;\;\;\;\;\;\;\;\;\;\;\;\;\;\;\;\;\;\;\; \end{array}$$

(34) $$\begin{array}{cc} \mathrm{Kurtosis} & =\frac{1}{\left|R\right|}\sum_{i=1}^{\left|R\right|}{\left(\frac{R_{i}-\mu }{\sigma }\right)}^{4}\;\;\;\;\;\;\;\;\;\;\;\;\;\;\;\;\;\;\;\;\;\;\;\;\;\;\;\;\;\;\;\;\;\;\; \end{array}$$

where $p_{k}$ denotes the normalized histogram bin for intensity level *k*, and ϵ is a small constant to prevent numerical instability.

#### 2.6.3. Texture Features

Texture features characterize spatial intensity relationships within vertebral marrow and provide quantitative measures of image heterogeneity. Because osteoporosis is associated with alterations in trabecular microarchitecture and marrow composition, texture descriptors may capture subtle structural variations that are not reflected by global intensity measures alone.

Two complementary texture analysis approaches were employed: Gray-Level Co-occurrence Matrix (GLCM) features and Local Binary Pattern (LBP) descriptors.

##### Gray-Level Co-Occurrence Matrix Features

Gray-Level Co-occurrence Matrices (GLCMs) [16] were computed from the masked image using multiple distances (d=1) and angles ($\theta \in \{0^{\circ },90^{\circ }\}$). From each GLCM, six Haralick texture features were extracted:

(35) $$\begin{array}{cc} \mathrm{Contrast} & =\sum_{i,j}{\left(i-j\right)}^{2}\;P\left(i,j\right) \end{array}$$

(36) $$\begin{array}{cc} \mathrm{Dissimilarity} & =\sum_{i,j}\left|i-j\right|\;P\left(i,j\right) \end{array}$$

(37) $$\begin{array}{cc} \mathrm{Homogeneity} & =\sum_{i,j}\frac{P(i,j)}{1+|i-j|} \end{array}$$

(38) $$\begin{array}{cc} \mathrm{ASM} & =\sum_{i,j}P{\left(i,j\right)}^{2}\;(\mathrm{Angular}\;\mathrm{Second}\;\mathrm{Moment}) \end{array}$$

(39) $$\begin{array}{cc} \mathrm{Energy} & =\sqrt{\mathrm{ASM}} \end{array}$$

(40) $$\begin{array}{cc} \mathrm{Correlation} & =\frac{\sum_{i,j}\left(i-\mu_{i}\right)\left(j-\mu_{j}\right)P\left(i,j\right)}{\sigma_{i}\sigma_{j}} \end{array}$$

Here, P(i,j) is the normalized GLCM and $\mu_{i},\sigma_{i}$ are the marginal means and standard deviations.

##### Local Binary Pattern Features

Local Binary Pattern [17] descriptors were additionally extracted to characterize local micro-texture patterns and intensity transitions within the vertebral ROI. Unlike GLCM features, which quantify pairwise spatial relationships, LBP encodes local neighborhood structure and provides robust characterization of fine-scale texture variation.

Local Binary Pattern histograms were computed using uniform encoding at multiple radii r∈{1,2,3}, with P=8r sampling points:

(41) $${\mathrm{LBP}}_{P,r}\left(x,y\right)=\sum_{p=0}^{P-1}s\left(g_{p}-g_{c}\right)\cdot 2^{p},\;s\left(z\right)=\left\{\begin{array}{cc} 1 & z\geq 0\\ 0 & \mathrm{otherwise} \end{array}\right.$$

A normalized histogram of LBP codes over the ROI was used as the feature vector for each scale.

#### 2.6.4. Structural Features

Structural features quantify local intensity transitions, edge characteristics, and directional organization within the vertebral ROI. These descriptors are intended to capture image patterns associated with trabecular organization, cortical boundary definition, and marrow–bone interfaces, all of which may be altered by osteoporosis-related structural degeneration.

To characterize structural organization, gradient-based operators and directional descriptors were employed, including Sobel gradients, Laplacian responses, Gaussian-smoothed representations, and Histogram of Oriented Gradients (HOG) features.

##### Edge and Gradient Features

To capture structural sharpness, boundary continuity, and local intensity transitions within vertebral trabecular regions, we extracted a set of edge- and gradient-based descriptors.

- Sobel operator: The Sobel filter estimates the local image gradient along the horizontal ($G_{x}$) and vertical ($G_{y}$) directions using a pair of 3×3 convolution kernels. The overall gradient magnitude is defined as $S=\sqrt{G_{x}^{2}+G_{y}^{2}}$, which highlights regions of high spatial variation—typically corresponding to trabecular boundaries or marrow–bone interfaces.
- Laplacian operator: The Laplacian [18] filter computes the second spatial derivative of the image, $L=\nabla^{2}I=\frac{\partial^{2}I}{\partial x^{2}}+\frac{\partial^{2}I}{\partial y^{2}}$. This operator responds strongly to rapid intensity changes and zero-crossings, emphasizing thin edges and fine texture variations.
- Gaussian smoothing: Prior to edge extraction, the image is smoothed by convolving it with a Gaussian kernel $G_{\sigma }$ to reduce noise sensitivity. The smoothed image $I_{G}=G_{\sigma }*I$ with σ=1 ensures that gradient and Laplacian computations emphasize genuine structural edges rather than random intensity fluctuations.

Together, these operators provide complementary information regarding local intensity variation, texture coarseness, and structural organization. Means and standard deviations of *S*, *L*, and $I_{G}$ within the ROI were extracted.

##### Histogram of Oriented Gradients Features

Directional gradient patterns were encoded using the Histogram of Oriented Gradients (HOG) [19]. Each patch was resized to 64×64 pixels before HOG computation with cell size (8,8) and block size (2,2). We retained:

(42) $$\begin{array}{c} \mu_{\mathrm{HOG}}=\frac{1}{\left|H\right|}\sum H_{i},\;\sigma_{\mathrm{HOG}}=\sqrt{\frac{1}{\left|H\right|}\sum {\left(H_{i}-\mu_{\mathrm{HOG}}\right)}^{2}} \end{array}$$

#### 2.6.5. Frequency Features

Frequency-domain analysis provides complementary information regarding image structure at different spatial scales. Whereas intensity and texture features primarily describe local image appearance, frequency-based descriptors characterize repetitive patterns, structural periodicity, and orientation-dependent texture information that may reflect trabecular organization.

##### Wavelet Transform Features

We applied a 2D Haar wavelet transform to decompose each image into frequency subbands:

(43) $$I\overset{\mathrm{DWT}}{\to }\left(cA,cH,cV,cD\right)$$

We extracted the mean and standard deviation of each of the detail components cH, cV, and cD.

##### Frequency-Orientation Features (Gabor Filters)

While wavelet features capture multi-resolution spatial frequency content, Gabor filters provide orientation-selective analysis and are particularly effective for characterizing anisotropic structural patterns. To model frequency-selective texture information, we applied Gabor [20] filters at frequencies f∈{0.1,0.2} and orientations $\theta \in \{0^{\circ },45^{\circ },90^{\circ },135^{\circ }\}$.

The real part of the filter response $G_{f,\theta }$ was extracted:

(44) Gf,θ(x,y)=ReI∗gf,θ

Mean and standard deviation over the ROI were recorded for each combination of (f,θ).

#### 2.6.6. Shape Features

Although osteoporosis primarily affects bone density and trabecular structure, chronic degenerative changes may also influence vertebral morphology. Therefore, shape descriptors were included to characterize geometric properties of the optimized vertebral ROIs.

Seven Hu [21] invariant moments $\phi_{i}$ were derived from raw spatial moments of the ROI:

(45) $$\mathrm{Hu}=[\phi_{1},\phi_{2},\ldots ,\phi_{7}]$$

These descriptors are invariant to translation, scale, and rotation, making them robust shape indicators.

For each vertebral level, all extracted features were concatenated into a single vertebral radiomic descriptor $f_{Lk}$. The final patient-level feature representation was assembled as:

(46) $$F=[f_{L1},f_{L2},f_{L3},f_{L4}]$$

This multi-scale radiomic representation integrates complementary information regarding vertebral marrow intensity, texture heterogeneity, structural organization, frequency-domain characteristics, and geometric morphology. The resulting feature vector serves as the input for subsequent osteoporosis-related classification and exploratory bone mineral density estimation.

#### 2.6.7. Feature-Group Importance Analysis

To quantify the contributions of different categories of radiomic descriptors to the classification decision, a post hoc feature-group importance analysis was performed on the trained classifier. The complete radiomic feature set was first partitioned into predefined feature groups according to the radiomic categories described in Section 2.6. These groups are mutually exclusive and collectively cover the entire feature representation.

Two complementary attribution techniques were employed: group-wise permutation importance and group-level Shapley value analysis.

##### Group-Wise Permutation Importance

Permutation importance is a model-agnostic technique for quantifying the contribution of input features by measuring the degradation in model performance after feature perturbation [22]. Let *f* denote the trained classifier and let G represent a feature group. Given a test feature matrix $X_{\mathrm{te}}\in R^{n\times d}$, all features belonging to G are jointly permuted across samples while the remaining feature groups remain unchanged,

$$X_{\mathrm{te}}^{\left(G\right)}\left[i,j\right]=\left\{\begin{array}{cc} X_{\mathrm{te}}\left[\pi \left(i\right),j\right], & j\in G,\\ X_{\mathrm{te}}\left[i,j\right], & j\notin G, \end{array}\right.\;\pi \in \mathrm{Sym}\left(n\right),$$

where *n* is the number of test samples and π denotes a random permutation. The permuted data are subsequently processed using the same preprocessing pipeline and evaluated by the trained classifier. The importance of each feature group is quantified as the degradation in the selected evaluation metric relative to the unpermuted baseline.

Permutation is performed at the feature-group level rather than on individual features to preserve the intrinsic correlation structure among descriptors derived from the same radiomic family. The procedure is repeated using multiple independent permutations, and the resulting importance scores are averaged to improve robustness.

##### Group-Level Shapley Values

To complement permutation importance, Shapley value analysis is performed at the feature-group level. Each feature group is regarded as a player in a cooperative game, where the contribution of a group is measured through its marginal contribution across all possible coalitions [23,24].

For a coalition S⊆N∖{g} and sample *x*, the coalition value is defined as

$$v\left(S,x\right)=\frac{1}{\left|B\right|}\sum_{b\in B}f\;\left(x_{S},b_{N\setminus S}\right),$$

where *B* denotes a representative background dataset. Feature groups belonging to *S* retain the values of the evaluated sample, whereas the remaining groups are substituted using the corresponding values from the background samples. The resulting samples are passed through the same preprocessing pipeline before being evaluated by the classifier.

The Shapley value of feature group *g* for sample *x* is computed as

$$\phi_{g}\left(x\right)=\sum_{S\subseteq N\setminus \{g\}}\frac{|S|!(|N|-|S|-1)!}{|N|!}\left[v(S\cup \{g\},x)-v(S,x)\right],$$

where *N* denotes the set of all feature groups. The global importance of each feature group is obtained by averaging the absolute Shapley values over all evaluation samples,

$$I_{g}=\frac{1}{n}\sum_{i=1}^{n}\left|\phi_{g}\left(x_{i}\right)\right|.$$

Together, these two analyses provide complementary perspectives on feature-group importance: permutation importance quantifies the loss in predictive performance when a feature group is disrupted, whereas Shapley values quantify the average contribution of each feature group to the classifier’s decision while accounting for interactions among feature groups.

### 2.7. Feature Standardization and Dimensionality Reduction

The proposed radiomic framework produces a high-dimensional feature representation composed of intensity, texture, structural, frequency-domain, and shape descriptors extracted from multiple vertebral levels. Because these features originate from heterogeneous measurement scales and statistical distributions, direct use of the raw feature vectors may bias machine learning models toward features with larger numerical ranges. Furthermore, the high dimensionality of the resulting feature space increases the risk of overfitting, particularly given the limited sample size available for training.

To address these challenges, a two-stage feature preprocessing strategy consisting of feature standardization and dimensionality reduction was employed.

#### 2.7.1. Feature Standardization

Prior to model training, each feature was standardized using z-score normalization:

(47) $$z_{i}=\frac{x_{i}-\mu }{\sigma }$$

where $x_{i}$ denotes the original feature value, μ is the mean of the feature computed from the training data, and σ is the corresponding standard deviation.

This transformation ensures that all features have zero mean and unit variance, thereby preventing variables with large numerical magnitudes from dominating the optimization process. Standardization is particularly important for distance-based and kernel-based learning algorithms such as Support Vector Machines (SVMs), where feature scaling directly influences the geometry of the feature space.

To avoid information leakage, the normalization parameters (μ and σ) were estimated exclusively from the training set within each experimental split and subsequently applied to the corresponding validation and test sets.

#### 2.7.2. Principal Component Analysis

Following standardization, Principal Component Analysis (PCA) [25] was applied to reduce feature redundancy and improve the stability of downstream machine learning models.

Let $X\in R^{N\times D}$ denote the standardized feature matrix, where *N* is the number of samples and *D* is the number of extracted radiomic features. PCA transforms the original feature space into a new set of orthogonal variables known as principal components:

(48) Z=XW

where W contains the eigenvectors of the covariance matrix of X, ordered according to decreasing eigenvalues.

The principal components were ranked according to the amount of variance explained:

(49) $$\mathrm{ExplainedVariance}\left(k\right)=\frac{\lambda_{k}}{\sum_{j=1}^{D}\lambda_{j}}$$

where $\lambda_{k}$ denotes the eigenvalue associated with the *k*-th principal component.

The number of retained components was determined using a cumulative explained variance threshold of 95%, such that

(50) $$\sum_{k=1}^{K}\frac{\lambda_{k}}{\sum_{j=1}^{D}\lambda_{j}}\geq 0.95$$

where *K* represents the number of retained principal components.

This strategy preserves the vast majority of information contained within the original radiomic feature space while substantially reducing dimensionality and feature redundancy. In addition, PCA mitigates multicollinearity among highly correlated radiomic features and reduces the risk of overfitting during model training.

As with feature standardization, PCA was fitted exclusively on the training data within each experimental split and subsequently applied to the corresponding validation and test sets to prevent information leakage.

The resulting low-dimensional feature representation served as the input for the osteoporosis assessment models described in the following section.

### 2.8. Osteoporosis Assessment Models

The extracted radiomic features were primarily used for osteoporosis classification, where subjects were categorized into osteoporotic and non-osteoporotic groups according to the corresponding DEXA-derived reference measurements. Because the proposed framework is intended as an opportunistic screening tool rather than a direct replacement for DEXA, osteoporosis classification was considered the primary clinical endpoint. In addition, quantitative bone mineral density (BMD) prediction was investigated as a secondary exploratory analysis to examine the relationship between MRI-derived radiomic features and continuous DEXA measurements.

#### 2.8.1. Experimental Protocol

To evaluate the robustness and generalizability of the extracted radiomic representations, two support-vector-machine-based classification frameworks were investigated: (1) a conventional Support Vector Classifier (SVC) and (2) the NeurodynamicSVMRBFTanh framework.

Model selection and hyperparameter optimization were performed using stratified five-fold cross-validation on the development cohort, ensuring that the class distribution was preserved across all folds. To mitigate the influence of class imbalance, random oversampling was applied exclusively to the training portion of each cross-validation fold after feature preprocessing, while the corresponding validation data remained untouched. Following hyperparameter selection, the classifiers were retrained on the complete training cohort and subsequently evaluated on an independent held-out test cohort consisting of 27 subjects. Subject-level partitioning was maintained throughout all experiments to ensure that no subject appeared simultaneously in both the training and testing sets, thereby preventing data leakage.

#### 2.8.2. Conventional Support Vector Classification

As a baseline classifier, a Support Vector Classifier (SVC) implemented in scikit-learn [26] was employed using a radial basis function (RBF) kernel. The RBF kernel enables nonlinear decision boundaries by mapping radiomic features into a higher-dimensional feature space and is widely used in medical image analysis due to its ability to model complex relationships in limited-sample settings. Hyperparameters, including the regularization parameter *C* and kernel bandwidth parameter γ, were optimized using grid-search cross-validation.

#### 2.8.3. Neurodynamic Kernel-Based Classification Framework

To investigate the robustness of the extracted radiomic features under an alternative kernel-based learning paradigm, the NeurodynamicSVMRBFTanh framework proposed by Wangkeeree et al. was also evaluated [27]. Unlike the conventional SVM, which is typically optimized using quadratic programming or other discrete iterative optimization algorithms, the proposed framework reformulates kernel SVM learning as a continuous-time neurodynamic system. Furthermore, the non-differentiable generalized pinball loss is replaced by a hyperbolic tangent smoothed approximation, yielding a continuously differentiable objective that can be efficiently optimized using gradient-based neural dynamics. The original study further establishes the equivalence between the equilibrium points of the neurodynamic system and the optimal solutions of the smoothed SVM formulation, together with theoretical guarantees on existence, uniqueness, convergence, and stability of the proposed dynamics. In this study, the framework was evaluated to determine whether these theoretical advantages translate into improved discrimination of osteoporosis from radiomic features.

#### 2.8.4. Exploratory BMD Regression

Although osteoporosis classification was the primary objective of this study, the feasibility of predicting quantitative bone mineral density (BMD) directly from MRI-derived radiomic features was also investigated. For this purpose, Support Vector Regression (SVR) with an RBF kernel was employed. The regression model is expressed as

(51) $$f\left(x\right)=\sum_{i=1}^{n}\left(\alpha_{i}-\alpha_{i}^{*}\right)K\left(x_{i},x\right)+b,$$

where K(·,·) denotes the RBF kernel and $\alpha_{i},\alpha_{i}^{*}$ represent the support-vector coefficients.

Model hyperparameters were optimized through cross-validation using the same strategy employed for the classification experiments. Regression performance was evaluated using standard error-based and correlation-based metrics and is reported as an exploratory analysis rather than a primary clinical endpoint. The objective of this analysis was to assess the extent to which MRI-derived radiomic features capture continuous variations in vertebral bone mineral density beyond categorical osteoporosis classification.

## 3. Results

### 3.1. Hierarchical Localization and Boundary Optimization

Prior to radiomic feature extraction, anatomically consistent identification of lumbar vertebral structures was required to ensure reliable characterization of trabecular bone tissue across subjects. To achieve this objective, a hierarchical localization framework was developed to progressively reduce the search space from the full sagittal MRI acquisition to individual vertebral bodies, followed by optimization-based boundary refinement.

**Figure 6:**
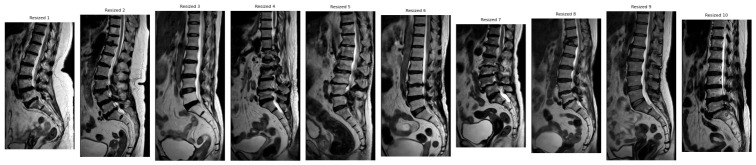
Representative preprocessed sagittal MR images after resizing and intensity normalization. Substantial variability in anatomy and image appearance highlights the need for robust localization strategies.

Figure 6 presents representative preprocessed sagittal MR images following resizing and intensity normalization. Considerable variability can be observed across subjects in terms of spinal curvature, vertebral morphology, field-of-view coverage, surrounding soft-tissue anatomy, and image contrast.

**Figure 7:**
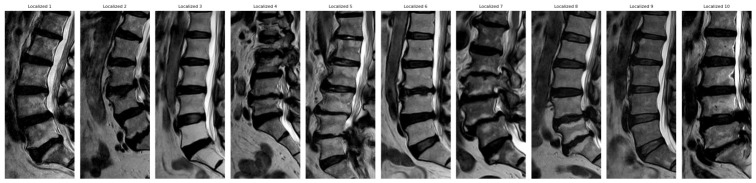
Automatically localized lumbar regions obtained using hierarchical template matching. The detected regions consistently encompass the target lumbar anatomy despite variations in spinal curvature and image appearance.

Figure 7 shows the output of the hierarchical localization stage. The framework successfully identified the lumbar spine across diverse image appearances and anatomical configurations. The detected regions consistently encompassed the target lumbar anatomy while excluding large portions of surrounding structures.

**Figure 8:**
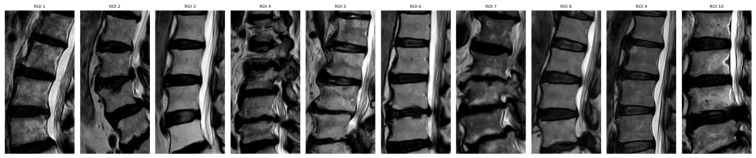
Standardized lumbar ROIs extracted following hierarchical localization. Anatomical alignment is substantially improved across subjects despite variations in spinal morphology.

Following localization, a standardized lumbar ROI was extracted from each subject, as shown in Figure 8. The extracted ROIs exhibited reduced positional variability and improved anatomical alignment across subjects.

**Figure 9:**
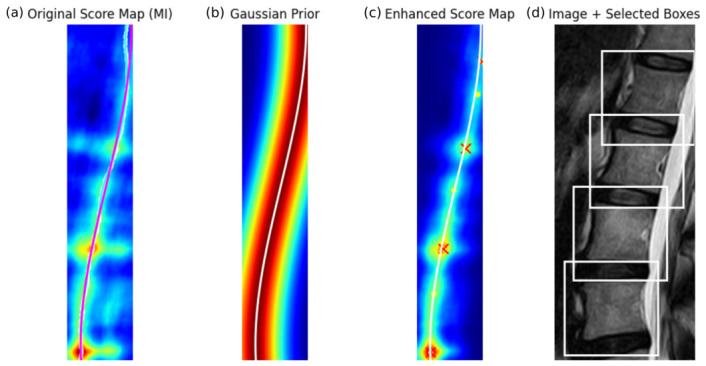
Anatomically guided vertebral detection framework. (**a**) Original mutual-information (MI) similarity map, where warmer colors indicate higher similarity scores. The magenta and cyan curves represent the estimated spinal trajectory and the fitted spinal centerline, respectively. (**b**) Gaussian anatomical prior centered on the estimated spinal trajectory, where red indicates higher prior probability and blue indicates lower prior probability. (**c**) Enhanced score map obtained by combining the similarity map with the anatomical prior. The white curve denotes the estimated spinal trajectory, the yellow circles indicate candidate vertebral locations, and the red crosses represent the optimized vertebral centers. (**d**) Final vertebral detections overlaid on the MRI image, where the white bounding boxes correspond to the detected L1–L4 vertebral regions.

Within the localized lumbar ROI, vertebral detection was performed using anatomically guided template matching. Figure 9 illustrates the detection procedure. The incorporation of the Gaussian anatomical prior concentrated candidate detections along the estimated spinal trajectory and suppressed anatomically implausible responses. The final detections corresponded to the L1–L4 vertebral bodies.

**Figure 10:**
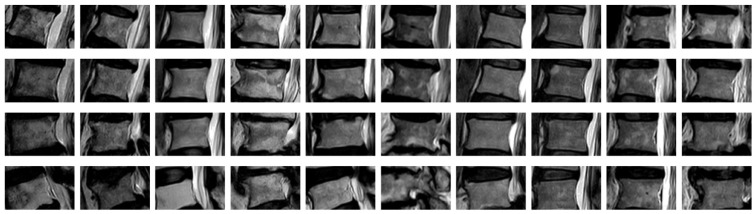
Representative vertebral body detections obtained from multiple subjects. Consistent anatomical coverage is achieved despite substantial inter-subject variability.

Representative vertebral detections from multiple subjects are shown in Figure 10. Consistent vertebral coverage was observed despite substantial variability in vertebral morphology, spinal curvature, and image appearance.

**Figure 11:**
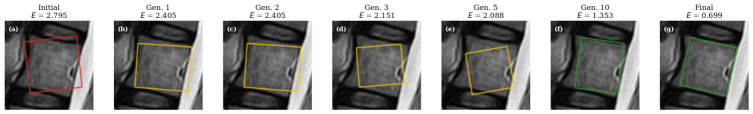
Progressive vertebral boundary refinement during Differential Evolution optimization. (**a**) Initial matched-template ROI (red bounding box; E=2.795). (**b**) Generation 1 (E=2.405). (**c**) Generation 2 (E=2.405). (**d**) Generation 3 (E=2.151). (**e**) Generation 5 (E=2.088). (**f**) Generation 10, approaching convergence (green bounding box; E=1.353). (**g**) Final optimized ROI after convergence (green bounding box; E=0.699). The red, yellow, and green bounding boxes denote the initial, intermediate, and converged ROI configurations, respectively.

The optimization-based boundary refinement stage further adjusted vertebral position, orientation, and dimensions. A representative example is shown in Figure 11. Starting from the initial localization, Differential Evolution iteratively refined the vertebral boundary according to the proposed objective function.

**Figure 12:**
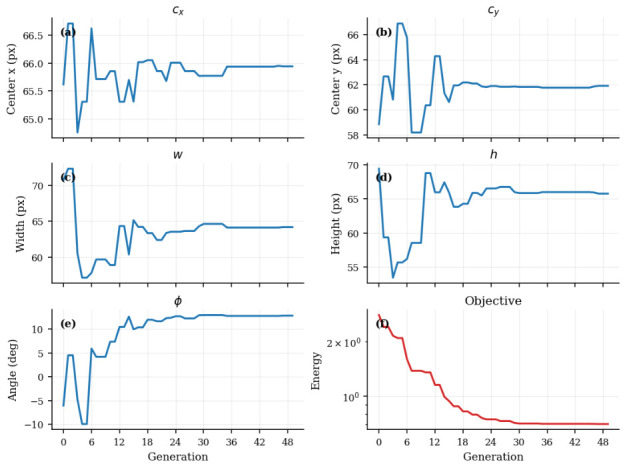
Convergence of the optimization variables during Differential Evolution. (**a**) Horizontal center coordinate ($c_{x}$). (**b**) Vertical center coordinate ($c_{y}$). (**c**) ROI width (*w*). (**d**) ROI height (*h*). (**e**) Rotation angle (ϕ). (**f**) Total objective energy.

**Figure 13:**
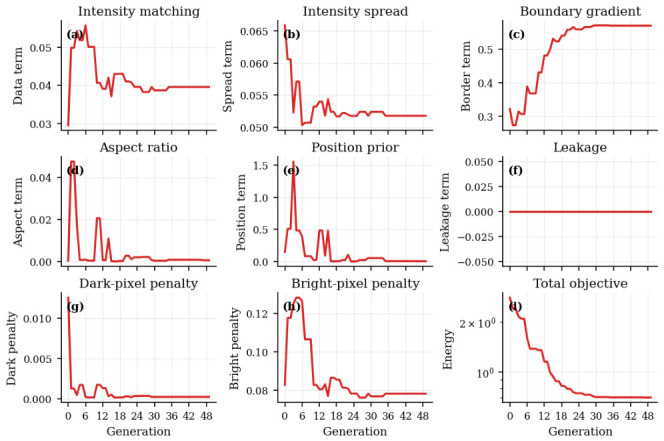
Evolution of the individual objective-function terms during optimization. (**a**) Intensity-matching term. (**b**) Intensity-spread term. (**c**) Boundary-gradient term. (**d**) Aspect-ratio regularization. (**e**) Position-prior term. (**f**) Leakage penalty. (**g**) Dark-pixel penalty. (**h**) Bright-pixel penalty. (**i**) Total objective energy.

Figure 11 illustrates the progressive refinement of the vertebral boundary throughout the Differential Evolution process. Starting from the approximate matched-template region, the rotated-rectangle ROI is iteratively adjusted so that its boundary converges toward the vertebral body, while the objective energy E(p) decreases monotonically from its initial value (E=2.795) to its converged value (E=0.699). The corresponding convergence behavior is summarized in Figure 12 and Figure 13: the geometric parameters $\left[c_{x},c_{y},w,h,\phi \right]$ stabilize within approximately 30 generations, and each individual energy component—data consistency, homogeneity, and boundary strength, together with the geometric and occupancy penalties—settles to a steady value, confirming stable convergence rather than oscillatory behavior.

This refinement is essential for reliable radiomic feature extraction. When features are computed directly from the unrefined matched-template region, the loosely fitted boundary encloses adjacent non-vertebral structures—such as intervertebral discs, the spinal canal, and surrounding background—whose intensity and texture patterns contaminate the extracted descriptors. Because radiomic features are highly sensitive to ROI definition, this contamination introduces variability that is unrelated to the vertebral body and undermines reproducibility. By constraining the ROI to align tightly with the vertebral boundary, the proposed refinement ensures that the extracted descriptors characterize only vertebral attributes rather than surrounding anatomy, thereby yielding more specific and reproducible radiomic representations.

### 3.2. Clinical VBQ Baseline

To provide a reference for comparison, vertebral bone quality (VBQ) assessment was evaluated against the DEXA-derived osteoporosis labels. Two orthopedic observers independently performed VBQ-based classification, and their averaged interpretation was additionally assessed. The resulting performance is summarized in Table 6.

The averaged VBQ assessment achieved an accuracy of 74.1%, sensitivity of 85.0%, specificity of 42.9%, and F1-score of 0.83. A total of 17 abnormal subjects were correctly identified, while 4 normal subjects were incorrectly classified as abnormal.

### 3.3. Radiomics-Based Osteoporosis Classification

The primary objective of this study was to evaluate whether MRI-derived radiomic features could identify subjects with abnormal bone quality using DEXA-derived reference labels. Classification performance was evaluated using both a conventional Support Vector Classifier (SVC) and the proposed NeurodynamicSVMRBFTanh framework across five MRI modality configurations (T1, T2, STIR, T1 + T2, and T1 + T2 + STIR).

#### 3.3.1. Cross-Validation Performance

Cross-validation results are summarized in Table 7. Among the conventional SVC models, T1-weighted MRI achieved the highest classification accuracy (69.4%), specificity (88.9%), and MCC (0.380).

For the NeurodynamicSVMRBFTanh framework, perfect sensitivity (100.0%) was achieved for the T1, T2, and STIR configurations. The highest MCC was obtained using T1-weighted MRI (0.529), while the highest AUC was observed for the T2 configuration (73.0%).

The multimodal configurations generally achieved higher sensitivity than the corresponding single-modality SVC models. The T1 + T2 + STIR NeurodynamicSVMRBFTanh model achieved an AUC of 72.4%, sensitivity of 95.2%, and MCC of 0.401.

#### 3.3.2. Independent Test Set Performance

Performance on the independent test set is summarized in Table 8. The NeurodynamicSVMRBFTanh framework achieved higher sensitivity and overall classification performance than the conventional SVC models.

Among all evaluated configurations, the NeurodynamicSVMRBFTanh model trained using T1-weighted MRI achieved the highest MCC (0.529), an accuracy of 85.2%, sensitivity of 100.0%, and an F1-score of 0.913. The T1 + T2 + STIR NeurodynamicSVMRBFTanh model also achieved an accuracy of 85.2% with a sensitivity of 95.2%.

The corresponding confusion matrix for the best-performing model is presented in Table 9. The model correctly identified all 21 abnormal subjects and produced no false-negative predictions. Two normal subjects were correctly classified, while four normal subjects were classified as abnormal.

Because the independent test set is small (n=27), point estimates for the best-performing model carry substantial uncertainty. Exact (Clopper–Pearson) 95% confidence intervals, computed from the confusion matrix in Table 9, are 83.9–100.0% for sensitivity (21/21), 4.3–77.7% for specificity (2/6), and 66.3–95.8% for accuracy (23/27). The wide interval for specificity in particular reflects the fact that only six normal subjects were available in the held-out test set; a single additional misclassified normal subject would shift the point estimate from 33.3% to 16.7%. These intervals should be considered when interpreting the reported point estimates and are reported here rather than for every modality/model combination in Table 8 because the small and unevenly distributed test set (n=6 normal, n=21 abnormal) makes per-configuration interval estimation of limited additional value.

Nevertheless, these findings should be interpreted in the context of the study design. The independent test cohort consisted of only 27 patients, of whom just 6 were classified as normal by DEXA; the reported 100% sensitivity therefore reflects perfect classification of 21 abnormal subjects, while the reported 33.3% specificity is based on only 6 normal subjects, for whom a single reclassified case would change the point estimate by nearly 17 percentage points (95% Clopper–Pearson CI: 4.3–77.7%, see Section 3). Given this small and class-imbalanced test set, together with the fact that hyperparameters and feature-reduction settings were selected via cross-validation on the training split before a single evaluation on the held-out set, some degree of optimism bias cannot be excluded, and the perfect sensitivity observed here should not be interpreted as an expectation of zero missed cases in a larger or external population.

The low specificity observed for the best-performing configuration also warrants explicit discussion of the false-positive burden it implies. At 33.3% specificity, roughly two-thirds of osteoporosis-negative patients in this cohort would be flagged for confirmatory evaluation. Whether this trade-off is acceptable depends on the clinical context in which the framework is deployed. In preoperative spine surgery screening specifically, a false negative—an osteoporotic patient proceeding to instrumentation without recognition of compromised bone quality—carries a materially higher downstream cost than a false positive, including risk of screw loosening, cage subsidence, proximal junctional kyphosis, and revision surgery, whereas a false positive results only in a confirmatory DEXA scan, a low-cost, low-risk, non-invasive test. This asymmetry supports prioritizing sensitivity over specificity for this specific use case, consistent with the design rationale of other high-sensitivity, lower-specificity opportunistic screening tools. However, a specificity in the 30–40% range implies a substantial confirmatory-testing burden if deployed unselectively across a general preoperative population with lower osteoporosis prevalence than the present cohort, and prospective evaluation in such a population, ideally with a larger and better-balanced test set, is needed before the operating point identified here can be considered clinically actionable rather than exploratory.

#### 3.3.3. Feature-Group Importance of Radiomic Descriptors

Although Table 8 demonstrates that the proposed NeurodynamicSVMRBFTanh achieves competitive discrimination between Normal and Abnormal (osteopenic/osteoporotic) vertebrae using T1-weighted radiomic descriptors, it does not reveal which categories of radiomic features contribute most to the classification performance. To investigate the relative importance of different image characteristics, the complete 546-dimensional T1 radiomic feature set was partitioned according to the feature groups defined in Section 2.6: Statistical (62 features), Texture (304 features), Structure (32 features), Frequency (120 features), and Shape (28 features). These groups are mutually exclusive and collectively account for all 546 radiomic features extracted from vertebral levels L1–L4 together with the pooled region-of-interest (ROI) features. The predictive contribution of each feature group was then evaluated independently using the same NeurodynamicSVMRBFTanh framework, allowing the relative importance of different radiomic characteristics for osteoporosis classification to be quantitatively compared.

**Figure 14:**
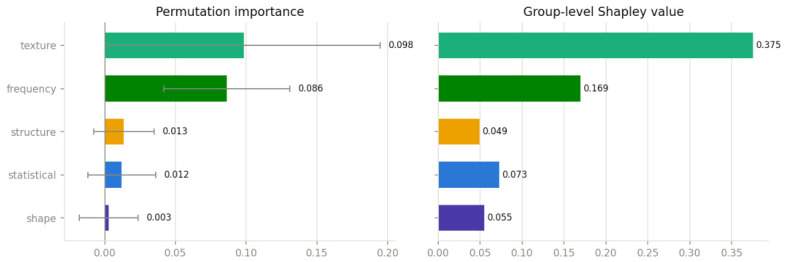
Feature-group importance for the selected T1 model (PinballProbSVC, τ=0.25). **Left**: Mean drop in held-out test AUC when a group’s columns are jointly permuted across patients (error bars: one standard deviation over 200 permutations). **Right**: Mean absolute exact Shapley value of each group on the model’s decision function, computed over all $2^{5}$ group coalitions. Colour is assigned per group and held fixed across both panels.

The resulting feature-group importance is summarized in Table 10 and Figure 14. Both group-wise permutation importance and group-level Shapley value analysis produced consistent rankings of the radiomic feature groups. Texture descriptors exhibited the largest contribution to the classification model, with the greatest decrease in predictive performance after permutation (ΔAUC=0.098±0.096, ΔMCC=0.143±0.137) and the highest mean absolute Shapley value (0.375). Frequency-domain descriptors ranked second, yielding a substantial reduction in classification performance (ΔAUC=0.086±0.045) and the second-largest Shapley importance (0.169).

In contrast, Statistical, Structure, and Shape descriptors exhibited considerably smaller importance scores across both attribution methods. Permutation of these feature groups resulted in only minor changes in classification performance, while their corresponding Shapley values remained substantially lower than those of the Texture and Frequency groups. Overall, both attribution methods consistently indicate that the classification model relies predominantly on texture- and frequency-related radiomic information, whereas first-order intensity, structural, and morphological descriptors provide comparatively limited contributions to the final classification decision.

### 3.4. Exploratory BMD Prediction

In addition to osteoporosis classification, MRI-derived radiomic features were evaluated for quantitative prediction of vertebral bone mineral density (BMD). Support vector regression (SVR) models were trained using five MRI modality configurations (T1, T2, STIR, T1 + T2, T1 + T2 + STIR) and evaluated on an independent 27-patient held-out test set for prediction of L1, L2, L3, L4, and Total Spine BMD. Hyperparameters (kernel, *C*, ε, γ) and a feature-reduction step (none, univariate *F*-test selection of 50/100 features, or PCA retaining 95% variance) were selected by 5-fold cross-validation on the training split; the selected configuration was then refit on the full training set and scored once on the held-out test set.

The independent test set performance is summarized in Table 11. Overall, multimodal MRI configurations generally achieved lower prediction errors than single-sequence models. For L2 BMD, the T1 + T2 configuration achieved the lowest prediction error (MAE $=0.111\;g/{\mathrm{cm}}^{2}$, RMSE $=0.144\;g/{\mathrm{cm}}^{2}$, MAPE =15.2%) and, notably, also the strongest linear agreement with ground truth of any target/modality pair (r=0.59, p=0.001). For Total Spine BMD, T1 + T2 likewise achieved the lowest overall prediction error (MAE $=0.142\;g/{\mathrm{cm}}^{2}$, RMSE $=0.167\;g/{\mathrm{cm}}^{2}$, MAPE =18.1%, r=0.39, p=0.052). For L1, L3, and L4 BMD, the T1 + T2 + STIR configuration achieved the lowest prediction errors among the evaluated models, with L1 and L3 BMD also reaching statistically significant correlation (r=0.41 and r=0.51 respectively, both p<0.05). Across all vertebral levels and modality configurations, MAPE ranged from 15.2% to 23.6%.

**Figure 15:**
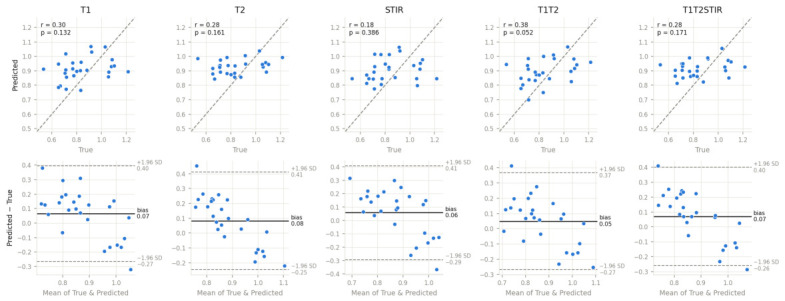
Total Spine BMD: Predicted vs. true scatter with identity line (**top row**) and Bland–Altman agreement plots (**bottom row**) for each of the five MRI modality configurations on the independent test set (n=27). Solid horizontal line: mean bias (predicted − true); dashed lines: 95% limits of agreement (bias ±1.96 SD). T1 + T2 shows the strongest correlation (r=0.39) and the narrowest limits of agreement (−0.27 to $+0.37\;g/{\mathrm{cm}}^{2}$) among the five configurations, consistent with its lowest MAE/RMSE/MAPE in Table 11.

Reporting MAE, RMSE, and MAPE in isolation, however, does not establish whether a model tracks patient-to-patient variation in BMD or has simply learned to predict values close to the sample mean; two models with identical RMSE can differ substantially in this respect. We therefore additionally report the Pearson correlation coefficient *r* between predicted and true BMD in Table 11 and perform a Bland–Altman agreement analysis for the primary clinical endpoint, Total Spine BMD, across all five modality configurations (Figure 15).

Two observations qualify the error metrics above. First, correlation with ground-truth BMD is weak-to-moderate across the majority of target/modality pairs: outside of the T1 + T2 and T1 + T2 + STIR configurations for L1–L3 BMD, most *r* values fall below 0.35 and do not reach statistical significance at α=0.05 (Table 11). For Total Spine BMD specifically, the best-performing configuration (T1 + T2) reaches only r=0.39 (p=0.052), i.e., borderline significance at n=27. Second, the Bland–Altman plots in Figure 15 reveal a small but consistent positive bias across every modality configuration (mean predicted − true BMD =+0.05 to $+0.08\;g/{\mathrm{cm}}^{2}$), indicating that the SVR models systematically over-predict BMD on average, i.e., tend to under-estimate bone loss severity. The scatter plots in the top row of Figure 15 further show that predicted BMD is compressed into a narrower range than the true BMD distribution, with the Bland–Altman difference sloping downward as the mean BMD increases (over-prediction at low true BMD, under-prediction at high true BMD)—a regression-to-the-mean pattern consistent with the modest *r* values, and one that MAE/RMSE/MAPE alone do not reveal.

These findings temper, without negating, the error-metric results above: T1 + T2 and T1 + T2 + STIR remain the most reliable modality configurations by every metric considered, including *r* and Bland–Altman limits of agreement, and several individual targets (L1–L3 BMD under multimodal inputs) achieve statistically significant, moderate correlation with ground-truth BMD. At the same time, given the modest sample size (n=27 for the held-out test set) and the systematic bias and regression-to-the-mean behavior identified above, we characterize these SVR results as an exploratory proof-of-concept for radiomic BMD regression rather than a clinically validated prediction tool, and note that a larger, ideally multi-site, cohort would be required to determine whether the observed correlations generalize.

## 4. Discussion

The present study proposed a fully automated and anatomically guided radiomics framework for opportunistic osteoporosis assessment from routine lumbar spine MRI. The framework integrates hierarchical vertebral localization, optimization-based ROI refinement, radiomic feature extraction, and machine learning-based prediction into a unified pipeline. The results demonstrate that routine lumbar MRI contains clinically meaningful information associated with bone quality and that this information can be exploited for automated osteoporosis assessment without additional imaging examinations.

### 4.1. Impact of Automated Vertebral Localization and ROI Refinement

Reliable vertebral localization and ROI definition are fundamental to MRI-based osteoporosis assessment because quantitative image features are highly dependent on the anatomical region from which they are extracted. Small variations in ROI placement can substantially alter texture, intensity, and structural descriptors, reducing measurement reproducibility and affecting downstream predictive performance. This limitation is particularly relevant for radiomics, where hundreds of quantitative features are extracted from each vertebral body, but it also affects conventional MRI-derived biomarkers such as VBQ and M-score, which rely on manually selected vertebral regions and observer-dependent signal intensity measurements.

To address these limitations, the proposed framework combines hierarchical vertebral localization with optimization-based ROI refinement to automatically identify anatomically consistent vertebral regions. The hierarchical localization strategy progressively narrows the search space from the entire lumbar spine to individual vertebral bodies, while the optimization framework refines the ROI by jointly considering image intensity, anatomical boundaries, geometric constraints, and vertebral morphology. Consequently, the extracted ROIs are more consistent across heterogeneous MRI examinations and less susceptible to operator-dependent variability.

The observed optimization results demonstrated stable convergence and consistent anatomical alignment across patients with varying spinal curvature, vertebral morphology, and image characteristics. By replacing manual ROI selection with a fully automated and anatomically guided procedure, the proposed framework improves the reproducibility and scalability of MRI-based osteoporosis assessment. Such automation is particularly advantageous for opportunistic screening, where large numbers of routine lumbar MRI examinations may be analyzed with minimal additional clinical workload.

### 4.2. Radiomics as an Alternative to Conventional MRI-Based Bone Quality Assessment

Radiomic analysis provides a substantially richer representation of vertebral bone quality than conventional MRI-derived biomarkers. The classification results demonstrate that radiomic descriptors extracted from routine lumbar MRI outperform the VBQ baseline, indicating that quantitative image analysis captures disease-related imaging characteristics beyond those represented by simple signal-intensity measurements.

Previous MRI-based osteoporosis studies have predominantly relied on intensity-derived biomarkers, such as VBQ scores, M-scores, or quantitative MRI parameters. Although these approaches have demonstrated promising diagnostic value, they are primarily based on global signal intensity and therefore provide only a limited characterization of vertebral bone quality. In contrast, radiomics simultaneously quantifies multiple complementary image properties, including first-order intensity statistics, texture, structural information, frequency-domain characteristics, and morphological descriptors.

The feature-group importance analysis provides further insight into the source of the predictive performance. Both group-wise permutation importance and group-level Shapley value analysis consistently identified texture and frequency descriptors as the dominant contributors to the classification model, whereas statistical, structural, and shape descriptors exhibited comparatively limited influence. This observation suggests that the discriminative information is primarily encoded in local spatial patterns and multi-scale frequency characteristics rather than global intensity measurements or gross vertebral morphology.

From a biological perspective, these findings are consistent with the pathophysiology of osteoporosis. Progressive deterioration of trabecular bone is characterized by thinning, perforation, and loss of connectivity within the trabecular network, producing increasingly heterogeneous marrow and bone patterns on MRI. Such microarchitectural alterations are naturally captured by texture descriptors, including GLCM and LBP features, while wavelet- and Gabor-based frequency features are sensitive to changes in spatial frequency content across multiple scales and orientations. Conversely, the overall shape of the vertebral body remains relatively preserved during the early stages of osteoporosis, explaining the comparatively small contribution of morphological descriptors.

These findings are also consistent with previous studies reporting associations between MRI texture features and DEXA-derived bone mineral density. The present results extend these observations by demonstrating, through complementary model attribution analyses, that radiomic features describing trabecular texture and frequency content constitute the principal source of predictive information. Collectively, the results suggest that radiomics provides a more comprehensive and biologically meaningful characterization of vertebral bone quality than conventional MRI-derived intensity biomarkers alone.

### 4.3. Performance of the NeurodynamicSVMRBFTanh Framework

Another important finding of this study is the consistently strong performance achieved by the NeurodynamicSVMRBFTanh framework. Compared with the conventional SVC, the proposed classifier demonstrated substantially higher sensitivity while maintaining competitive overall classification performance, making it particularly suitable for screening-oriented osteoporosis assessment.

From a clinical perspective, high sensitivity is essential for opportunistic osteoporosis screening, where the primary objective is to identify individuals at increased fracture risk before clinically significant complications occur. Consequently, false-negative predictions are generally more detrimental than false-positive referrals, as missed osteoporosis cases may delay diagnosis and subsequent treatment. The ability of the proposed framework to identify nearly all abnormal subjects therefore represents a clinically desirable operating characteristic.

The improved performance may be attributed to both the characteristics of the radiomic feature space and the optimization strategy employed by the NeurodynamicSVMRBFTanh framework. The extracted radiomic representation integrates heterogeneous feature families, including statistical, texture, structural, frequency-domain, and shape descriptors, resulting in a high-dimensional feature space with substantial inter-feature correlations and complex nonlinear relationships. Furthermore, the feature-group importance analysis demonstrated that classification is driven primarily by texture and frequency-domain descriptors, suggesting that discriminative information is encoded in subtle trabecular image patterns rather than simple intensity differences. Learning an effective decision boundary under such conditions is therefore inherently challenging.

Unlike the conventional SVC, which is optimized using the hinge loss, the NeurodynamicSVMRBFTanh framework employs a hyperbolic tangent smoothed generalized Pinball loss within a continuous-time neurodynamic optimization framework. The resulting continuously differentiable objective enables gradient-based optimization while retaining the robustness of the generalized Pinball loss to noisy and ambiguous samples. In addition, the original formulation provides theoretical guarantees on the existence, uniqueness, convergence, and stability of the optimization dynamics, providing a rigorous mathematical foundation for the learning process [27]. These characteristics make the framework well suited for learning from heterogeneous radiomic representations and likely contribute to its improved performance compared with the conventional SVC.

Nevertheless, these findings should be interpreted in the context of the study design. The independent test cohort consisted of only 27 patients, which limits the statistical power for comparing classification models. Although the observed improvement demonstrates the potential of the NeurodynamicSVMRBFTanh framework for osteoporosis assessment from radiomic features, further validation using larger, independent, multi-center cohorts is warranted to confirm its robustness and generalizability.

### 4.4. Influence of MRI Modality

Among the evaluated imaging configurations, T1-weighted MRI consistently demonstrated the strongest single-modality performance. This observation is biologically plausible because osteoporosis is associated with progressive changes in vertebral marrow composition, including increased marrow adiposity and alterations in trabecular microarchitecture, both of which influence T1-weighted signal characteristics.

The superior performance of T1-weighted imaging is consistent with previous investigations reporting strong associations between marrow-sensitive MRI biomarkers and bone quality measurements. These findings suggest that routine T1-weighted lumbar spine MRI may already contain sufficient information for clinically useful osteoporosis assessment without requiring specialized imaging protocols.

Multimodal MRI combinations generally improved robustness and, in several experiments, enhanced predictive performance. The integration of T1-, T2-, and STIR-weighted information likely provides complementary information regarding marrow composition, tissue hydration, structural heterogeneity, and pathological changes. Nevertheless, the strong performance achieved using T1-weighted MRI alone suggests that opportunistic screening may be feasible using imaging sequences that are already routinely acquired during clinical spine examinations.

To help contextualize whether a 15.2–23.6% MAPE represents a clinically meaningful level of agreement, it is useful to compare it against known sources of measurement variability in BMD assessment itself. Reported short-term precision error for lumbar spine DEXA, i.e., repeat scans of the same patient on the same scanner, is typically on the order of 1–2% coefficient of variation, corresponding to a least significant change of approximately 3–5% [28]. This figure, however, reflects only within-device reproducibility; across different units of the same manufacturer, larger discrepancies are expected. In a multi-center proficiency study using a certified European Spine Phantom, Hologic devices, the same manufacturer and Discovery-W model used at our institution, showed accuracy errors of approximately 5–6% relative to the phantom’s true reference values and an inter-device precision (CVsd) of 0.78% across vertebral levels [29], notably larger than same-scanner precision but still well below the error observed in the present study. Cross-modality comparisons show still larger discrepancies: in a phantom study directly comparing CT-derived volumetric BMD against a calibrated density reference, relative measurement errors for conventional quantitative CT ranged from approximately 3.7% to 9.7% depending on vertebral level, though dual-source dual-energy CT reduced this to below 1% [30]. Against this context, the 15.2–23.6% MAPE observed for MRI radiomics-based BMD estimation in the present study exceeds the intra-device precision error of DEXA, the inter-device error observed even between units of the same DEXA manufacturer, and the inter-modality error reported between CT-based volumetric BMD and a calibrated reference standard. This comparison reinforces the framing adopted above: in its current form, MRI radiomics-based BMD estimation carries error substantially larger than the measurement variability that clinicians already tolerate between BMD-assessment devices and modalities, and should therefore be regarded as a risk-stratification signal rather than an interchangeable substitute for DEXA-derived BMD.

### 4.5. Comparison with Recent MRI-Based Osteoporosis Assessment Methods

Recent MRI-based osteoporosis assessment has progressively evolved from simple intensity-derived biomarkers toward increasingly automated and quantitative image analysis. Early approaches, including the Vertebral Bone Quality (VBQ) score and M-score, primarily relied on manually measured vertebral marrow signal intensity and demonstrated meaningful associations with DEXA- or QCT-derived bone mineral density (BMD), fragility fractures, and osteoporosis diagnosis [3,6,8]. More recent studies have further extended MRI-based assessment by combining information from multiple MRI sequences or by exploiting quantitative parameters derived from synthetic MRI [7,9].

With the growing interest in artificial intelligence, recent work has also explored automated MRI-based osteoporosis assessment using radiomics and deep learning. Zhao et al. [13] integrated deep-learning vertebral segmentation with radiomic feature extraction for osteoporosis classification, while Galbusera et al. [14] combined MRI-derived radiomics, deep learning, radiographic measurements, and clinical information for both BMD estimation and osteoporosis assessment. Vara et al. [11] demonstrated that texture-based radiomic features extracted from manually defined vertebral ROIs were associated with DEXA-derived BMD, highlighting the potential of MRI radiomics for quantitative bone quality assessment.

Table 12 summarizes representative MRI-based osteoporosis assessment methods and positions the proposed framework within the current literature. Compared with previous intensity-based biomarkers, the proposed framework combines fully automated vertebral localization, optimization-based ROI refinement, and multi-domain radiomic feature extraction within a unified analysis pipeline. Furthermore, unlike previous radiomics-based studies, the present work provides feature-group importance analysis using permutation importance and Shapley values, improving the interpretability of the extracted radiomic representation. In addition to osteoporosis classification, the framework also explores quantitative BMD estimation, enabling both categorical and continuous assessment of vertebral bone quality.

Overall, the proposed framework extends existing MRI-based opportunistic osteoporosis assessment by integrating automated vertebral analysis, anatomically consistent radiomic feature extraction, explainable machine learning, and exploratory BMD prediction into a single end-to-end pipeline.

### 4.6. Potential Integration into Clinical Radiology Workflow

The proposed framework is designed to complement, rather than replace, existing clinical radiology practice by enabling opportunistic osteoporosis screening during routine lumbar spine MRI examinations. Unlike conventional MRI-derived biomarkers such as VBQ and M-score, which require manual vertebral localization and signal intensity measurements, the proposed framework performs vertebral localization, ROI refinement, radiomic feature extraction, and osteoporosis risk assessment automatically without requiring user interaction. As a result, no additional imaging sequences, radiation exposure, or changes to existing acquisition protocols are required.

Following image acquisition, the automated analysis can be performed in the background before radiologist interpretation. The generated osteoporosis risk assessment may be incorporated into the radiology report or presented as a decision-support alert within the picture archiving and communication system (PACS). Rather than serving as a standalone diagnostic tool, the framework is intended to assist radiologists by identifying patients with potentially compromised bone quality who may benefit from confirmatory DEXA examination or further clinical evaluation.

This workflow has the potential to improve opportunistic osteoporosis detection in patients undergoing lumbar MRI for unrelated indications, such as degenerative spine disease, chronic low back pain, or preoperative assessment. By leveraging routinely acquired clinical images, the proposed framework may facilitate earlier osteoporosis identification while reducing observer variability and preserving the existing radiology workflow without increasing examination time or patient burden.

### 4.7. Limitations and Future Work

Several limitations should be acknowledged. First, the study was conducted using a relatively limited single-center dataset acquired from a single-vendor 1.5T MRI system with a consistent imaging protocol, which may limit the generalizability of the proposed framework. Although an independent test cohort was employed, external validation across multiple institutions, scanner vendors, field strengths, and acquisition protocols is necessary to confirm the robustness and clinical applicability of the proposed approach. Furthermore, the class distribution in the present cohort may not fully reflect that of routine opportunistic screening populations, where osteoporosis prevalence is generally lower. Although stratified cross-validation and training-set oversampling were adopted to mitigate class imbalance during model development, model performance may differ when applied to larger and more imbalanced real-world clinical populations.

Second, the proposed framework operates on two-dimensional sagittal MRI examinations rather than fully volumetric three-dimensional data. Future studies may investigate whether volumetric vertebral analysis can provide additional structural information and further improve predictive performance.

Third, only handcrafted radiomic descriptors were evaluated in this work. While handcrafted features provide interpretability and computational efficiency, deep learning representations may capture complementary imaging characteristics that are not explicitly represented by conventional radiomic descriptors. Future work may also explore hybrid frameworks that integrate handcrafted radiomics with deep learning features to leverage the strengths of both approaches.

Future research will focus on multicenter validation using heterogeneous MRI datasets, prospective evaluation in routine clinical screening populations, integration of radiomics and deep learning representations, and extension toward broader skeletal health assessment applications, including fracture risk prediction and vertebral compression fracture evaluation.

## 5. Conclusions

This study presented a fully automated, anatomically guided radiomics framework for opportunistic osteoporosis assessment from routine lumbar spine MRI, integrating hierarchical vertebral localization, optimization-based ROI refinement, multi-domain radiomic feature extraction, and machine learning-based classification within a single end-to-end pipeline. Unlike prior MRI-based approaches that depend on manually placed ROIs and intensity-derived biomarkers such as VBQ or M-score, the proposed framework removes manual intervention from vertebral identification and ROI definition while capturing texture, structural, frequency-domain, and morphological characteristics of bone quality that extend well beyond global signal intensity.

Classification results showed that the extracted radiomic representation outperformed the VBQ baseline, and feature-group importance analysis, based on permutation importance and Shapley values, identified texture and frequency-domain descriptors as the dominant contributors to this performance—a pattern consistent with the trabecular microarchitectural deterioration characteristic of osteoporosis. Among the evaluated classifiers, the NeurodynamicSVMRBFTanh framework achieved the strongest and most sensitive performance, an operating characteristic well suited to screening applications where missed cases carry greater clinical cost than false referrals. T1-weighted MRI alone provided the strongest single-modality performance, while multimodal T1/T2/STIR combinations improved robustness, indicating that opportunistic screening is feasible using sequences already acquired during routine spine examinations. Exploratory regression analysis further showed that MRI radiomics correlates with DEXA-derived BMD, though individual-level prediction accuracy remains insufficient to replace densitometry directly; the framework is therefore best positioned as a risk-stratification and referral tool rather than a substitute for DEXA.

Relative to existing MRI-based methods, including recent automated radiomics and deep-learning pipelines, the proposed framework is distinguished by combining full automation, multi-domain radiomic characterization, and model-level interpretability—a combination not previously demonstrated for MRI-based osteoporosis screening. These properties support integration into existing radiology workflows without additional imaging sequences, radiation exposure, or protocol changes, enabling at-risk patients to be flagged for confirmatory DEXA testing directly from studies already acquired for other clinical indications.

These findings should be interpreted in light of the study’s single-center dataset of modest size, two-dimensional sagittal analysis, and reliance on handcrafted radiomic features. Future work will focus on multicenter and larger-scale validation, volumetric and deep-learning-based feature representations, and extension of the framework to related applications such as fracture risk prediction and vertebral compression fracture assessment.

## Figures and Tables

**Table 1:** MRI acquisition characteristics of the study dataset.

Parameter	Value
MRI Scanner	Philips Ingenia 1.5T
Sequences	Sagittal T1W, T2W, STIR
Body Part Examined	LSPINE
Slice Thickness	4.0 mm
Spacing Between Slices	4.4 mm
Pixel Spacing	0.3472×0.3472–0.5294×0.5294 mm
Image Size	400×400 to 1024×1024 pixels
Number of Subjects	167

**Table 2:** Distribution of subjects according to WHO osteoporosis diagnostic criteria derived from DEXA T-scores.

Class	Diagnostic Group	T-Score Criterion	*n*	(%)
0	Normal	T>−1.0	26	15.57
1	Osteopenia	−2.5<T≤−1.0	66	39.52
2	Osteoporosis	T≤−2.5	75	44.91
Total	–	–	167	100.00

**Table 3:** Binary classification grouping used for osteoporosis assessment.

Binary Class	Included WHO Categories	*n*
Non-osteoporosis	Normal + Osteopenia	92
Osteoporosis	Osteoporosis	75
Total	–	167

**Table 4:** Parameter settings used for anatomically guided vertebral detection.

Parameter	Value	Role
*k*	0	Statistical threshold coefficient
$d_{\min}$	0.10	Minimum peak distance ratio
$q_{\mathrm{row}}$	0.80	Quantile threshold for high-confidence responses
$p_{\mathrm{row}}$	2.0	Response amplification exponent
$\sigma_{y}$	6.0	Gaussian smoothing parameter
$w_{\mathrm{MA}}$	9	Moving-average window size
$d_{\mathrm{poly}}$	3	Polynomial curve degree
$p_{\mathrm{mass}}$	1.0	Response weighting exponent
$r_{x}$	0.15	Relative width of anatomical prior ($\sigma_{x}=r_{x}w$)
α	1.0	Anatomical prior strength
$\lambda_{\mathrm{line}}$	1.0	Alignment consistency weight
$\lambda_{\mathrm{spacing}}$	1.0	Inter-vertebral spacing regularization weight
$\lambda_{\mathrm{spread}}$	0.3	Vertebral separation weight
$\lambda_{\mathrm{score}}$	1.0	Template matching confidence weight

**Table 5:** Parameter settings used for optimization-based vertebral ROI refinement.

Parameter	Value	Role
$x_{t}$	0.30	Target vertebral intensity
$a_{0}$	1.0	Expected aspect ratio
α	0.1	Data consistency weight
β	20.0	Homogeneity weight
γ	2.0	Boundary alignment weight
δ	7.0	Aspect ratio regularization weight
η	0.2	Positional prior weight
ϵ	0.01	Leakage penalty weight
$\zeta_{d}$	100.0	Dark occupancy penalty weight
$\zeta_{b}$	10.0	Bright occupancy penalty weight
$T_{d}$	0.20	Dark intensity threshold
$T_{b}$	0.40	Bright intensity threshold

**Table 6:** VBQ-based binary classification performance from two orthopedic observers and their averaged interpretation.

Metric	VBQ A	VBQ B	Mean VBQ
TP	17	16	17
FP	4	4	4
TN	3	3	3
FN	3	4	3
Precision (%)	81.0	80.0	81.0
Sensitivity (%)	85.0	80.0	85.0
Specificity (%)	42.9	42.9	42.9
NPV (%)	50.0	42.9	50.0
Accuracy (%)	74.1	70.4	74.1
F1-score	0.83	0.80	0.83
F0.5-score	0.82	0.80	0.82
F2-score	0.84	0.80	0.84

**Table 7:** Cross-validation performance of traditional SVC and NeurodynamicSVMRBFTanh across MRI modality configurations. Best values are highlighted.

Model	Modality	AUC (%)	ACC (%)	Sens (%)	Spec (%)	MCC
SVC	T1	69.8	**69.4**	50.0	**88.9**	0.380
SVC	T2	60.6	57.7	57.1	58.3	0.154
SVC	STIR	59.5	55.9	61.9	50.0	0.118
SVC	T1 + T2	67.2	63.8	66.7	61.1	0.284
SVC	T1 + T2 + STIR	68.1	65.0	71.4	58.3	0.302
NeurodynamicSVMRBFTanh	T1	69.1	66.7	**100.0**	33.3	**0.529**
NeurodynamicSVMRBFTanh	T2	**73.0**	50.0	**100.0**	0.0	0.000
NeurodynamicSVMRBFTanh	STIR	59.5	50.0	**100.0**	0.0	0.000
NeurodynamicSVMRBFTanh	T1 + T2	71.2	61.1	95.2	27.8	0.322
NeurodynamicSVMRBFTanh	T1 + T2 + STIR	72.4	63.9	95.2	33.3	0.401

**Table 8:** Independent test set performance of VBQ, traditional SVC, and NeurodynamicSVMRBFTanh. Best values are highlighted.

Method	Modality	AUC (%)	ACC (%)	Sens (%)	Spec (%)	F1-Score	MCC
VBQ	Mean VBQ	–	74.1	85.0	42.9	0.83	–
SVC	T1	69.8	62.9	50.0	**88.9**	0.64	0.380
SVC	T2	60.6	59.3	57.1	58.3	0.59	0.154
SVC	STIR	59.5	55.6	61.9	50.0	0.58	0.118
SVC	T1 + T2	67.2	70.4	71.4	66.7	0.72	0.381
SVC	T1 + T2 + STIR	68.1	74.1	76.2	66.7	0.76	0.442
NeurodynamicSVMRBFTanh	T1	69.1	**85.2**	**100.0**	33.3	**0.913**	**0.529**
NeurodynamicSVMRBFTanh	T2	**73.0**	77.8	**100.0**	0.0	0.875	0.000
NeurodynamicSVMRBFTanh	STIR	59.5	77.8	**100.0**	0.0	0.875	0.000
NeurodynamicSVMRBFTanh	T1 + T2	71.2	81.5	95.2	33.3	0.89	0.322
NeurodynamicSVMRBFTanh	T1 + T2 + STIR	72.4	**85.2**	95.2	50.0	0.91	0.401

**Table 9:** Confusion matrix of the best-performing NeurodynamicSVMRBFTanh model using T1-weighted MRI on the independent test set.

		Predicted Class		
		Normal	Abnormal	Total
Actual	Normal	TN = 2	FP = 4	6
	Abnormal	FN = 0	TP = 21	21
	Total	2	25	27

**Table 10:** Feature-group importance for PinballProbSVC(τ=0.25) on the T1 held-out test set (n=27). Permutation columns report the mean ± standard deviation drop in test AUC/MCC over 200 block shuffles; the Shapley column reports the mean absolute exact group-level Shapley value on the decision function.

Feature Group	ΔAUC (Perm.)	ΔMCC (Perm.)	Mean $|\phi_{g}|$ (Shapley)
Texture	0.098±0.096	0.143±0.137	0.375
Frequency	0.086±0.045	0.035±0.075	0.169
Statistical	0.012±0.024	0.040±0.049	0.073
Shape	0.003±0.021	0.006±0.020	0.055
Structure	0.014±0.021	0.006±0.022	0.049

**Table 11:** Independent test set performance for BMD prediction using SVR across MRI modality configurations (n=27). Lower MAE/RMSE/MAPE indicate better error; higher *r* indicates stronger linear agreement with the ground-truth BMD. Best MAE/RMSE/MAPE for each target is highlighted; *r* values marked * are significant at p<0.05 (Pearson).

MRI Input	Target	MAE	RMSE	MAPE (%)	*r*
T1	L1 BMD	0.142	0.170	18.3	0.27
T1	L2 BMD	0.136	0.158	18.0	0.43 *
T1	L3 BMD	0.180	0.204	23.6	0.31
T1	L4 BMD	0.173	0.200	21.0	0.27
T1	Total Spine BMD	0.155	0.178	19.8	0.30
T2	L1 BMD	0.145	0.172	18.9	0.25
T2	L2 BMD	0.136	0.169	19.0	0.40 *
T2	L3 BMD	0.153	0.187	20.4	0.36
T2	L4 BMD	0.162	0.201	19.8	0.20
T2	Total Spine BMD	0.157	0.184	20.7	0.28
STIR	L1 BMD	0.147	0.176	18.6	0.15
STIR	L2 BMD	0.129	0.157	16.8	0.31
STIR	L3 BMD	0.174	0.199	21.5	0.18
STIR	L4 BMD	0.170	0.205	19.7	0.21
STIR	Total Spine BMD	0.165	0.185	20.5	0.18
T1 + T2	L1 BMD	0.129	0.166	17.0	0.38
T1 + T2	L2 BMD	**0.111**	**0.144**	**15.2**	0.59 *
T1 + T2	L3 BMD	0.174	0.205	23.1	0.40 *
T1 + T2	L4 BMD	0.173	0.206	21.2	0.02
T1 + T2	Total Spine BMD	**0.142**	**0.167**	**18.1**	**0.39**
T1 + T2 + STIR	L1 BMD	**0.127**	**0.162**	**16.3**	0.41 *
T1 + T2 + STIR	L2 BMD	0.125	0.150	16.3	0.49 *
T1 + T2 + STIR	L3 BMD	**0.157**	**0.175**	**20.3**	0.51 *
T1 + T2 + STIR	L4 BMD	**0.155**	**0.193**	**18.9**	0.32
T1 + T2 + STIR	Total Spine BMD	0.155	0.179	20.1	0.28

**Table 12:** Comparison of representative recent MRI-based opportunistic osteoporosis assessment methods.

Study	MRI	Method	Automatic ROI	Radiomics	Osteoporosis Assessment	BMD Estimation
Ehresman et al. [3]	T1	VBQ	✗	✗	✓	✗
Roch et al. [6]	T1/T2/STIR	VBQ	✗	✗	✓	✓
Xue et al. [7]	T1	Simplified VBQ (T12)	✗	✗	✓	✓
Saad et al. [8]	T1	M-score	✗	✗	✓	✓
Chang et al. [9]	Synthetic MRI	Quantitative MRI	✗	✗	✓	✓
Vara et al. [11]	T1/T2	Radiomics	✗	✓	✗	✓
Zhao et al. [13]	mDixon MRI	DL-Segmented Radiomics	✓	✓	✓	✓
Galbusera et al. [14]	T1/T2	Radiomics + DL	✓	✓	✓	✓
Proposed	T1/T2/STIR	Radiomics + ML	✓	✓	✓	✓

✓ = supported; ✗ = not supported.

## Data Availability

The data presented in this study are available on request from the corresponding author. The data are not publicly available due to ethical, legal, and privacy restrictions associated with patient medical data and institutional regulations governing data sharing.
